# Development of an Untargeted LC-MS Metabolomics Method with Postcolumn Infusion for Matrix Effect Monitoring in Plasma and Feces

**DOI:** 10.1021/jasms.3c00418

**Published:** 2024-02-21

**Authors:** Pingping Zhu, Anne-Charlotte Dubbelman, Christie Hunter, Michele Genangeli, Naama Karu, Amy Harms, Thomas Hankemeier

**Affiliations:** † Metabolomics and Analytics Centre, Leiden Academic Centre for Drug Research, Leiden University, Leiden 2333 CC, Netherlands; ‡ Institute for Risk Assessment Sciences, Utrecht University, Utrecht 3584 CM, The Netherlands; § SCIEX, Redwood City, California 94065, United States

**Keywords:** untargeted metabolomics, method development, matrix effect, postcolumn infusion, plasma, feces

## Abstract

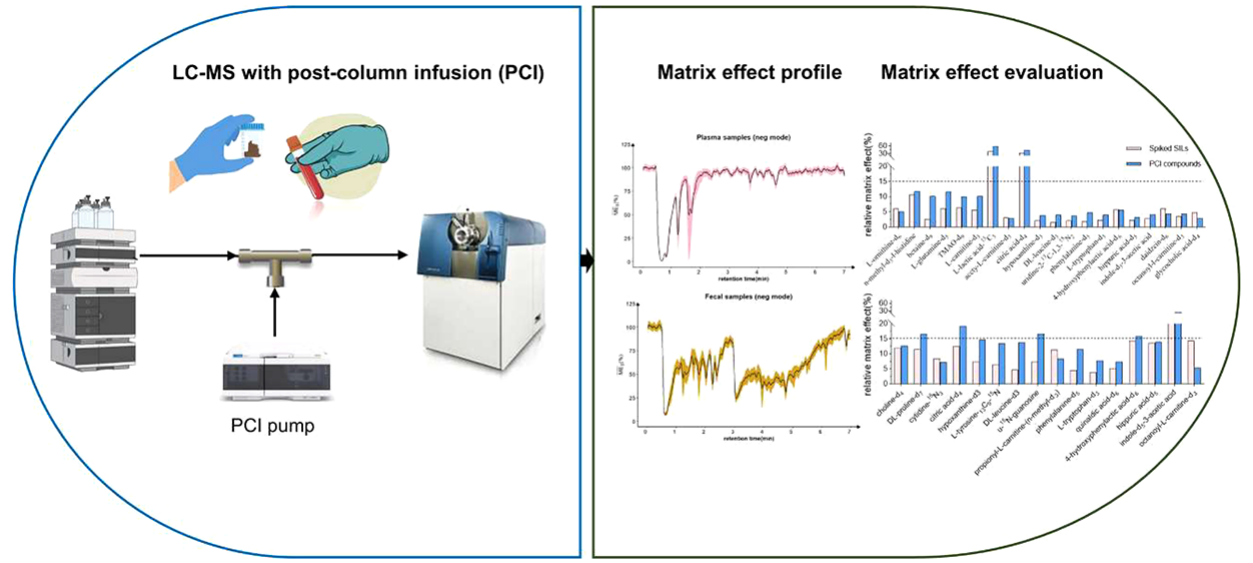

Untargeted metabolomics based on reverse phase LC-MS (RPLC-MS) plays a crucial role in biomarker discovery across physiological and disease states. Standardizing the development process of untargeted methods requires paying attention to critical factors that are under discussed or easily overlooked, such as injection parameters, performance assessment, and matrix effect evaluation. In this study, we developed an untargeted metabolomics method for plasma and fecal samples with the optimization and evaluation of these factors. Our results showed that optimizing the reconstitution solvent and sample injection amount was critical for achieving the balance between metabolites coverage and signal linearity. Method validation with representative stable isotopically labeled standards (SILs) provided insights into the analytical performance evaluation of our method. To tackle the issue of the matrix effect, we implemented a postcolumn infusion (PCI) approach to monitor the overall absolute matrix effect (AME) and relative matrix effect (RME). The monitoring revealed distinct AME and RME profiles in plasma and feces. Comparing RME data obtained for SILs through postextraction spiking with those monitored using PCI compounds demonstrated the comparability of these two methods for RME assessment. Therefore, we applied the PCI approach to predict the RME of 305 target compounds covered in our in-house library and found that targets detected in the negative polarity were more vulnerable to the RME, regardless of the sample matrix. Given the value of this PCI approach in identifying the strengths and weaknesses of our method in terms of the matrix effect, we recommend implementing a PCI approach during method development and applying it routinely in untargeted metabolomics.

## 1 Introduction

Untargeted metabolomics is a powerful approach that has demonstrated great potential in exploring metabolic changes in health and disease conditions.^1−3^ Its application has extended beyond biomedical research to fields such as food, agricultural, and environmental studies,^4−6^ thereby making it a highly valuable tool for diverse scientific research. One of the most widely used techniques for untargeted metabolomic analysis is ultraperformance liquid chromatography coupled to mass spectrometry (UPLC-MS).^7^ Among the different types of UPLC-MS, reverse phase LC-MS (RPLC-MS) is the most popular choice for UPLC-MS due to its versatility, robustness, stability, and good retention of semipolar to nonpolar metabolites.^8^ As the popularity of untargeted metabolomics has increased, researchers have focused on standardizing the development process of this method, especially when aiming at semiquantitative analysis beyond qualitative compound discovery and screening. Parameters such as sample extraction, LC-MS system selection and setup, quality management, and analysis batch design have been extensively studied and advised upon.^9−11^ However, some critical factors required to develop a reliable untargeted RPLC-MS platform are either easily overlooked or still under discussion for standardization. Those factors include the optimization of the injection solvent and the sample injection amount. In the sample preparation process of untargeted methods, an evaporation and reconstitution step is typically performed to allow for flexibility in modifying the injection solvent composition and the sample loading amount. This step is important to prevent mismatch between the mobile phase and injection solvent and to balance the challenge of maximizing the metabolome coverage, minimizing signal saturation and reducing the matrix effect.^12,13^ The reconstitution solvent can affect peak shape and metabolite coverage in RPLC for untargeted analysis of small molecules,^14,15^ which emphasizes the importance of investigating the injection solvent during the development of a RPLC-MS method. Another critical parameter is the injection amount, which was reported to impact the data quality and repeatability in terms of overloading, signal saturation and feature missingness.^16^ Therefore, a systematic investigation of the injection amount is also critical when developing an RPLC-MS method.^13^

The investigation of reconstitution solvents typically involves assessing the peak shape and signal intensity of representative metabolites.^14,15^ When investigating the injection amount, serially diluted standards or samples are commonly used to evaluate signal linearity.^13,16^ In order to maintain signal linearity in high-resolution MS, techniques such as dynamic ion transmission control (ITC) and automatic gain control (AGC) have been developed to modulate the ion amounts in various regions of the MS system. The ITC technique, implemented in all QTOF systems from SCIEX, modulates the ion current scan by scan to ensure it remains within the dynamic range of the detection system. For trap-based MS instruments from Thermo Fisher, AGC is employed to automatically regulate the ion amount in the ion-trap by adjusting the fill time for every scan. These techniques not only extend the dynamic range of the MS system but also offer insights into the ion transmission status through the MS system. Recently, they have been employed as effective approaches to investigate ion transmission during method development with high-resolution MS,^17^ making them promising readouts for the optimization of the sample injection amount to avoid the risk of signal nonlinearity.

Another challenge in untargeted metabolomics is method performance assessment and validation. Despite the recommendations for addressing quality assurance and quality control challenges,^9,18^ there is currently no consensus on the performance validation of untargeted methods during the development phase. However, it has been recommended that in addition to monitoring signal drift and repeatability with pooled quality control (QC) samples, an untargeted method can be validated in a targeted way with representative metabolites.^19^ This strategy has been widely applied in untargeted metabolomics research to validate the parameters of linearity, precision, recovery, and accuracy with selected endogenous metabolites.^9,20−22^ However, in these studies, serially diluted pooled QC samples were commonly used to evaluate the linearity, leading to the dilution of both the targeted analyte and the matrix, which reduces the reliability of this strategy.^19^ The matrix effect has been regarded as one of the most significant challenges of LC-MS methods, especially when analyzing complex biological matrices.^23,24^ Therefore, the matrix effect is another widely discussed factor in untargeted metabolomics because of its impact on reproducibility, linearity, selectivity, accuracy, and sensitivity.^25^

The phenomenon of the matrix effect was first reported in 1993 by Tang and Kebarle, who observed that the signal of an analyte ionized by the electrospray ionization (ESI) source can be strongly affected by the presence of other electrolytes in the solution.^26^ Although the exact mechanism of how the matrix effect occurs is still unclear, it is commonly assumed that the coeluted matrix components can affect the ionization of an analyte by preventing or competing with the analyte to gain charge, increasing the surface tension of the charged droplet, interfering with the stability of charged analytes in the gas phase, and/or coprecipitating with the analyte.^27^ To overcome the matrix effect in LC-MS, two main strategies have been proposed: matrix effect reduction and matrix assessment/correction. Matrix effect reduction can be achieved through extensive sample cleaning procedures, enhanced LC separation efficiency, sample dilution, or adopting alternative MS ionization sources other than ESI.^25,28−30^

Matrix effect assessment can mainly be achieved by postextraction spiking and postcolumn infusion (PCI) of compounds.^24,25^ The postextraction spike method was proposed by Matuszewski et al. to quantitatively assess the matrix effect by comparing the response in neat standard solution samples with that in postextraction spiked samples. They also introduced the terms absolute matrix effect (AME) and relative matrix effect (RME) to describe the matrix effect, where AME is the response ratio of an analyte at a given concentration spiked in postextraction biological samples compared to neat solution samples and RME is the variability of AME among different lots of biological samples.^30^ Following the introduction of the postextraction spiking method, the term matrix factor (MF) was introduced as a quantitative measure of the matrix effect that shares the same concept with AME.^31^ The MF was later applied in accordance with the European Medicine Agency (EMA) guideline released in 2011 for the ME assessment in bioanalytical method validation.^32^ According to the guideline, the MF variability (RME) should not exceed 15%. In contrast to the postextraction spiking method, which assesses the matrix effect at specific time points, the PCI technique was proposed by Bonfiglio et al. as a method for matrix effect assessment across the entire LC chromatogram.^33^ In PCI, a compound is constantly infused into the MS after joining the column effluent using a T-connector. This enables the infusion profile of the compound to be observed across the entire chromatogram with the injection of a matrix sample and a blank sample, allowing for real-time monitoring of the matrix effect. Due to this advantage, PCI has been utilized in targeted analysis for matrix effect evaluation and correction for small molecules and drugs in urine and plasma samples.^34−37^

Unfortunately, although multiple strategies have been proposed for reducing and assessing the matrix effect, few are applicable to untargeted LC-MS methods. In these methods, simple and unbiased sample preparation is required to broaden the metabolite coverage and, in order to represent a compromise that accommodates most classes, the LC separation is typically not tailored for specific compound classes.^12,38^ Therefore, in terms of matrix effect reduction, aside from switching to an ionization source other than ESI, sample dilution is the only applicable approach in untargeted metabolomics. For the matrix effect assessment and correction, the postextraction spiking method is more suitable for targeted metabolomics due to the requirement of authentic standards. Hence, PCI is recommended as a more appropriate tool for matrix effect evaluation in untargeted metabolomics,^29,39^ but only few studies about its application have been reported.^40,41^ Although stable isotope labeling has also been applied to matrix effect evaluation in untargeted metabolomics, this technique is limited to specific sample types like yeast, cells, or plants due to the requirement of a globally labeled growth medium.^42−44^

In this study, we developed an RPLC-MS untargeted metabolomics method suitable for the measurement of plasma and feces, taking into account both matrix diversity and the growing popularity of fecal metabolome studies. Initially, we optimized the injection solvent and injection amount for both matrices and validated the optimized platform in a targeted manner. To evaluate the matrix effect of plasma and feces alongside other performance parameters (precision, accuracy, and recovery) and guarantee the reliability of the linearity, stable isotopically labeled standards (SILs), instead of endogenous metabolites, were used in the validation. These SILs are well distributed in terms of class, retention time, physicochemical properties, and abundance according to their endogenous analogues. By validating our method with these SILs, we have gained insight into the analytical performance. Additionally, we augmented this untargeted method with a PCI approach for matrix effect monitoring, which offers the advantage of overall matrix effect evaluation of plasma and fecal samples. This allows us to identify the strengths and weaknesses of our method in terms of the matrix effect, ensuring better data reliability in untargeted metabolomics. The successful application of PCI for matrix effect monitoring in this untargeted metabolomics method strongly suggests that this approach can be widely implemented in the development and routine analysis of an LC-MS untargeted method.

## 2 Methods

### 2.1 Chemicals and Materials

LC-MS-grade acetonitrile (ACN) and methanol (MeOH) were purchased from Actu-all chemicals (Randmeer, The Netherlands). Methyl *tert*-butyl ether (MTBE, ≥99.8%) and sodium hydroxide were purchased from Sigma-Aldrich (St. Louis, Missouri, United States). Formic acid (FA) was purchased from Biosolve B.V. (Valkenswaard, Netherlands), and hydrochloric acid (37% solution in water) was purchased from Acros Organics (Geel, Belgium). Purified water was obtained from a Milli-Q PF Plus system (Merck Millipore, Burlington, Massachusetts, United States). Most chemical standards and stable isotopically labeled standards (SILs) were purchased from CDN Isotopes (C/D/N Isotopes Inc., Quebec, Canada), Cambridge Isotope Laboratories (Tewksbury, MA, USA), and TRC (Toronto Research Chemicals, Toronto, Canada). Table S1 provides the supplier details of all standards. Pooled EDTA plasma was obtained from Innovative Research (Peary Court Novi, Michigan, United States), pooled male and female ETDA plasma was purchased from Sanquin (Sanquin, Amsterdam, The Netherlands), and ETDA plasma from individual donors was purchased from BioIVT (Westbury, New York, United STates). Fecal samples were collected from four healthy adults, including three female volunteers and one male volunteer (age range: 23–35 years old).

### 2.2 Solution Preparation

#### 2.2.1 Preparation of Calibrant Solutions

The stock solutions of 28 authentic SILs were prepared at different concentrations in appropriate solvents (Table S1). For certain SILs, ammonium hydroxide or hydrochloric acid was added to improve solubility. Standard mixture solutions were prepared by mixing 21 (plasma validation) or 16 (feces validation) SILs. Those mixtures were serially diluted with water to obtain working calibration solutions at 9 (plasma) or 11 (feces) concentration levels (see Tables S2 and S3). Stock solutions and standard mixtures were stored at −80 °C until use, and calibration solutions were freshly prepared before experiments.

#### 2.2.2 Preparation of Internal Standards and Reconstitution Solution

Fludrocortisone-*d*_5_, glucose-^13^C_6_-*d*_7_, caffeine-*d*_9_, and valine-*d*_8_ were added as internal standards (IS) for signal drift monitoring. Detailed information on those IS is shown in Table S1. Four IS were spiked in plasma validation, while three IS (except glucose-^13^C_6_-*d*_7_) were spiked for fecal validation. Cortisone-*d*_8_ in water with 0.1% FA was prepared as the reconstitution solution.

#### 2.2.3 Preparation of Solutions of PCI Compounds

Leucine-enkephalin, fludrocortisone, 5-fluoroisatin, caffeine-^13^C_3_, and 3-fluoro-dl-valine were selected as the PCI compounds considering their physical properties, ionization behaviors, availability, and cost. All the PCI compounds were prepared with water, MeOH, or water/MeOH (1:1, v/v) (Table S1). The postcolumn infusion mixture solution was prepared with water/ACN (1:1, v/v). In the positive mode, the PCI comprised leucine-enkephalin, fludrocortisone, 5-fluoroisatin, and caffeine-^13^C_3_, while in negative mode it included leucine-enkephalin, fludrocortisone, and 3-fluoro-dl-valine. Table S4 provides the final concentrations of each PCI compound in the mixture solutions.

### 2.3 Sample Preparation

#### 2.3.1 Plasma Sample Preparation

Protein precipitation was used to prepare plasma samples. Aliquots of 25 μL of plasma were mixed with 10 μL of IS working solution and quenched with 90 μL of ice-cold MeOH. All samples were then vortex mixed (1 min, high speed), incubated on ice (20 min), and centrifuged (15 min, 15 600 g, 4 °C). Afterward, 100 μL of supernatant from each sample was transferred to 1.5 mL Eppendorf tubes and evaporated to dryness in a SpeedVac (Labcono, Kansas City, Missouri, United States). The residuals were reconstituted in 75 μL of water with 0.1% FA, vortex mixed (1 min, high speed), and centrifuged (5 min, 2300 g, 4 °C). Finally, 70 μL of the supernatant was transferred to autosampler vials, and 1 μL was injected into the LC-MS.

During method development, extracted plasma samples were reconstituted in 50 μL of 0.1% FA in water with 0%, 10%, or 20% of ACN (v/v/v) to optimize reconstitution solvent and in 50, 75, or 150 μL of 0.1% FA in water to evaluate sample dilution factors (DF) of 1:2, 1:3, and 1:6 (v/v), respectively. Of those samples, 1 and 2 μL was injected to compare injection volumes.

For method validation, calibration lines (*n* = 3) were created using pooled EDTA plasma with 10 μL of spiked calibration working solutions. Precision was evaluated at each concentration level from the calibration lines. Pooled EDTA plasma, pooled male EDTA plasma, pooled female EDTA plasma, and one individual EDTA plasma were used as four different plasma samples for recovery, accuracy, and matrix effect evaluation. For recovery, plasma samples were prepared by spiking 10 μL of calibration working solutions to get concentrations at low (cal4), medium (cal6), and high (cal8) levels before extraction and after drying. The samples spiked before extraction were also used for the evaluation of accuracy. Samples for the matrix effect evaluation were prepared by spiking 10 μL of calibration working solution at three concentration levels in plasma and matrix-free (solvent) samples after drying.

#### 2.3.2 Fecal Sample Preparation

##### 2.3.2.1 Final Sample Preparation Procedure

Fecal samples were stored at −20 °C immediately after collection. Samples were thawed at ambient temperature and homogenized as proposed by Hosseinkhani et al. (involving stirring, sonication for 5 min, and vortex mixing for 10 min),^45^ with the adjustment that 1 mL of water per gram of sample was added at the start to improve homogenization. The homogenized and aliquoted samples (around 2 g per tube) were stored at −80 °C for more than 48 h before lyophilization. Freeze-drying was conducted overnight (20 h, 4 mbar, −110 °C) with a CHRIST Alpha 3–4 LSCbasic freezer-dryer (Martin Christ, Germany) and 20 mg (±0.3 mg) aliquots of lyophilized sample were weighed and stored at −80 °C until extraction.

Liquid–liquid extraction (LLE) was performed as recommended by Hosseinkhani et al.,^45^ whereby the starting amount was adapted to 20 mg of dried feces, considering the added water and limited sample size of clinical samples. Added volumes for extraction were changed accordingly. Briefly, 108 μL of ice-cold MeOH (5.4 μL mg^–1^ dried feces) and 36 μL of ice-cold water (1.8 μL mg^–1^ dried feces) were added to 1.5 mL Eppendorf tubes with 20 mg of freeze-dried feces, followed by vortex mixing (2 min). Then, 60 μL of ice-cold MTBE (3 μL mg^–1^ dried feces) was added, followed by vortex mixing (2 min) and centrifugation (15 min, 16 000 g, 4 °C,). Next, 140 μL of the supernatant was transferred to clean tubes. Phase separation was induced by adding 84 μL of ice-cold MTBE (4.2 μL mg^–1^ dried feces) and 100 μL of ice-cold water (5 μL mg^–1^ dried feces). Then samples were remixed for 2 min and kept at 4 °C for 10 min to obtain better protein precipitation. After centrifugation (20 min, 16 000 g, 4 °C), 90 μL of the aqueous layer was transferred to 1.5 mL Eppendorf tubes and evaporated to dryness. The remainder of the aqueous layer was saved for other analyses. The dried residuals were reconstituted in 50 μL of reconstitution solution, resulting in the ratio of dried feces to reconstitution solvent being around 1:8 (mg/v) (calculation details are provided in Table S5). All the samples were vortex mixed (5 min) and centrifuged (5 min, 16 000 g, 4 °C) before being transferred to autosampler vials, and 1 μL was injected into the LC-MS.

##### 2.3.2.2 Sample Preparation for Reconstitution Solvent, Dilution Factor, and Injection Volume Comparison

Pooled fecal samples from three individuals were used to optimize the reconstitution solution, dilution factor, and injection volume for feces. The individual samples were homogenized separately, and equal amounts were aliquoted, pooled, mixed, and homogenized. Freeze-dried feces (50 mg) from the pooled sample was aliquoted and extracted with MTBE/MeOH/water (3.6:2.7:3.4, v/v/v). After LLE, the aqueous layer was transferred to 1.5 mL Eppendorf tubes and evaporated in the SpeedVac. Dried fecal extracts were reconstituted in 300 μL of 0.1% FA in water with 0%, 10%, or 20% of ACN (v/v/v) to evaluate the reconstitution solvent and in 150 or 300 μL of 0.1% FA in water to evaluate sample DF of 1:3 and 1:6 (mg/v), respectively. Of those samples, 1 and 2 μL aliquots were injected to optimize injection volume.

##### 2.3.2.3 Sample Preparation for Validation

A pooled sample from four donors was used to build the calibration line and assess precision and recovery. Samples from each individual were used for accuracy and matrix effect evaluation. Calibration lines were constructed by spiking the calibrant solution at each level to the samples after LLE extraction. Samples for recovery evaluation were prepared by spiking the calibrant solution in fecal samples to get concentrations at low (cal4) and high (cal10) concentration levels before LLE extraction and after drying. The samples spiked after drying were also used for the evaluation of accuracy. Samples for the matrix effect evaluation were prepared by spiking calibrant solutions in fecal and matrix-free (solvent) samples to get concentrations at low (cal4), medium (cal7), and high (cal10) levels after drying. The final sample preparation procedure for feces was followed for the steps of extraction, reconstitution, and injection.

### 2.4 Method Validation

#### 2.4.1 Linearity

The linearity of selected SILs in both plasma and feces was evaluated by calibration lines (*n* = 3). The calibration lines of the SILs applied in plasma and feces were designed based on the concentration levels of their endogenous analogues (Figure S1). The calibration points and ranges of SILs after being spiked in plasma and feces are presented in Tables S2 and S3.

#### 2.4.2 Precision, Accuracy, and Recovery

Precision was expressed as the relative standard deviation (RSD) of the peak area for each calibration point in three calibration lines. Accuracy and recovery were evaluated at different concentration levels with four samples. The accuracy was calculated by dividing the calibration line back-calculated concentration by the nominal concentration at each level. The recovery was calculated as the ratio of the SILs peak area obtained in the samples spiked before extraction and after drying at each concentration level.

#### 2.4.3 Matrix Effect

The absolute matrix effect (AME) and relative matrix effect (RME) were both evaluated with four different plasma and fecal samples. The AME was assessed by calculating the ratio of peak area obtained in the matrix (postextraction) and matrix-free sample (solvent sample). The RME was expressed as the RSD of the AME.

### 2.5 LC-MS Conditions and Postcolumn Infusion Setup

Analysis was performed on a reverse phase UPLC-MS untargeted platform. The platform consisted of a Shimadzu Nexera X2 LC system coupled to a TripleTOF 6600 mass spectrometer (SCIEX, Foster City, California, United States) with an electrospray ionization source (ESI) that operated at both positive and negative ion modes. The ESI source parameters were set as follows: spray voltage ±4.5 kV, capillary temperature 400 °C, sheath gas 40, auxiliary gas 40, and curtain gas 45. Data were acquired under the full scan mode over the *m*/*z* range of 60–800 Da. The LC separation was carried out using a Waters Acquity UPLC HSS T3 column (1.8 μm, 2.1 mm × 100 mm) with the oven temperature maintained at 40 °C. The mobile phase A was 0.1% FA in water, and the mobile phase B was 0.1% FA in ACN. With a flow rate of 0.4 mL min^–1^, the gradient started at 100% A and was held for 0.5 min, then B linearly increased to 20% over 2.5 min and continuously increased to 98% from 2.5 to 7.5 min. This condition was maintained for 4.5 min, then returned to 100% A in 0.1 min, at which time the column was equilibrated for 3 min, resulting in a 15 min run time per analysis. The autosampler temperature was set at 10 °C. To decelerate the contamination of the MS, the LC flow was diverted to waste at 7 min of the gradient by an external valve (Valco Instruments, United States). During the analysis, the PCI compounds were continuously pumped by a binary Agilent 1260 Infinity pump (Agilent Technologies, Santa Clara, California, United States) at a flow rate of 20 μL min^–1^ and combined to the LC flow with a T-piece (IDEX, PEEK Tee, 0.02 Thru hole, F-300) before entering the ESI source.

### 2.6 Data Processing

The raw data were obtained using Analyst TF software 1.7.1 (SCIEX) and processed using SCIEX OS (version 2.1, SCIEX) and PeakView (version 2.2, SCIEX) software. Extracted ion chromatograms (EICs) were obtained for each compound, including PCI compounds with an *m*/*z* window of 0.02 Da. A maximum mass error of 5 ppm was applied for peak integration of all the compounds, and the retention times of endogenous compounds were verified using authentic standards. Count conversion factor plots were viewed in PeakView. This option can be enabled by closing the PeakView software, copying the “Instrument Utilities.dll” file from the “C:\Program Files\AB SCIEX\PeakView 2\bin” folder to the “C:\Program Files\AB SCIEX\PeakView 2\Help” folder, and restarting the software. Then, when opening a datafile and extracting the TOF MS TIC, navigate to the “Help” menu in PeakView software, click on the “Instrument Utilities.dll” and select “Plot Count Conversion Factors”. The PCI infusion profiles were generated by smoothing the extracted EIC data using the simple moving average function (SMA, *n* = 20) in R (version 4.2.1). To generate matrix effect profiles (MEPs), the matrix effect of each time point was calculated as reported in the literature.^41^ This calculation involved dividing the EIC response (*R*) of each PCI compound in the matrix sample by that in the blank sample (eq 1) and smoothing accordingly.
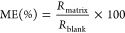
1
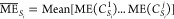
2
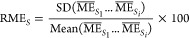
3

To evaluate the RME among four individuals, the absolute matrix effect signal of one sample () was first calculated by averaging the matrix effect of all the PCI compounds (C^1^_–_C^*j*^) in that sample (eq 2). Then, the RME among four individual samples (RME_*S*_) was calculated as the RSD of  (eq 3). The calculated RME profile from certain samples was used to predict RME for targets detected in those samples based on their retention times.

## 3 Results and Discussion

### 3.1 Analytical Performance Evaluation of the Developed Method

Before method validation, we optimized the reconstitution solvent and injection amount for both plasma and fecal samples, as detailed in the “reconstitution solvent and injection amount optimization” section in the Supporting Information. In summary, 0.1% FA in water outperformed solutions with 10% and 20% ACN and was chosen as the final injection solvent, considering the peak shape for the metabolites of interest. After signal intensity comparison and detector saturation checking through dynamic ion transmission control (ITC), dilution factors DF3 (1:3, v/v) and DF8 (1:8, mg/v) were selected for plasma and fecal samples, respectively, with an injection volume of 1 μL. In the analytical performance evaluation, we validated the untargeted method in both plasma and fecal samples. The dynamic range, precision, accuracy, recovery, and matrix effect were evaluated with selected SILs.

#### 3.1.1 Plasma Validation

The linearity range and precision are summarized in Table S7. We obtained good linearity (*R*^2^ > 0.98) with a wide range for 19 of 21 SIL targets. The inclusion and exclusion criteria for the calibration points were based on the acceptable residual error (<20%) compared to the nominal concentration. At least five consecutive concentration levels were required to build a calibration curve. DCA-*d*_4_ could not form a calibration curve, as only three continuous concentration levels were within acceptable criteria, probably caused by its solubility issue as described in the “reconstitution solvent and injection amount optimization” section in the Supporting Information. Good precision (RSD < 15%) was achieved for most of the acceptable concentration levels. The accuracy, recovery, and matrix effect were assessed with three concentration levels (low, medium, high). However, only medium and high concentrations were evaluated for *n*-methyl-d_3_-l-histidine, indole-*d*_5_-3-acetic acid, and GCA-*d*_4_ because the low concentration fell below the detection limit. None of them was evaluated for DCA-*d*_4_ due to the unavailability of the calibration curve.

Good recoveries were obtained for 20 SILs (within 80–120%), except for TMAO-*d*_9_, which exhibited a recovery of around 65% at low and medium concentration levels (Figure S5a). The accuracy between the back-calculated concentration and the nominal concentration was within 67–122% for all SIL targets, except for citric acid-*d*_4_, which had an accuracy close to 200% (Figure S5b). The imprecise accuracy of citric acid-*d*_4_ was caused by the varying levels of citric acid in the different plasma samples. We observed that the citric acid level in the pooled plasma used for creating the calibration curve was much higher than the other plasma we used for accuracy evaluation. Therefore, with an identical spiked concentration of citric acid-*d*_4_, a higher response was observed in the plasma with lower endogenous citric acid due to lower rate of ion suppression. When applying the calibration line built with suppressed signal to the samples that suffered less ion suppression, the back-calculated concentrations will be higher than the spiked ones due to the higher observed response, resulting in the inaccuracy of citric acid-*d*_4_. The impact of ion suppression on accuracy emphasizes the importance of matrix effect evaluation, especially the relative matrix effect among samples.

**Figure 1:**
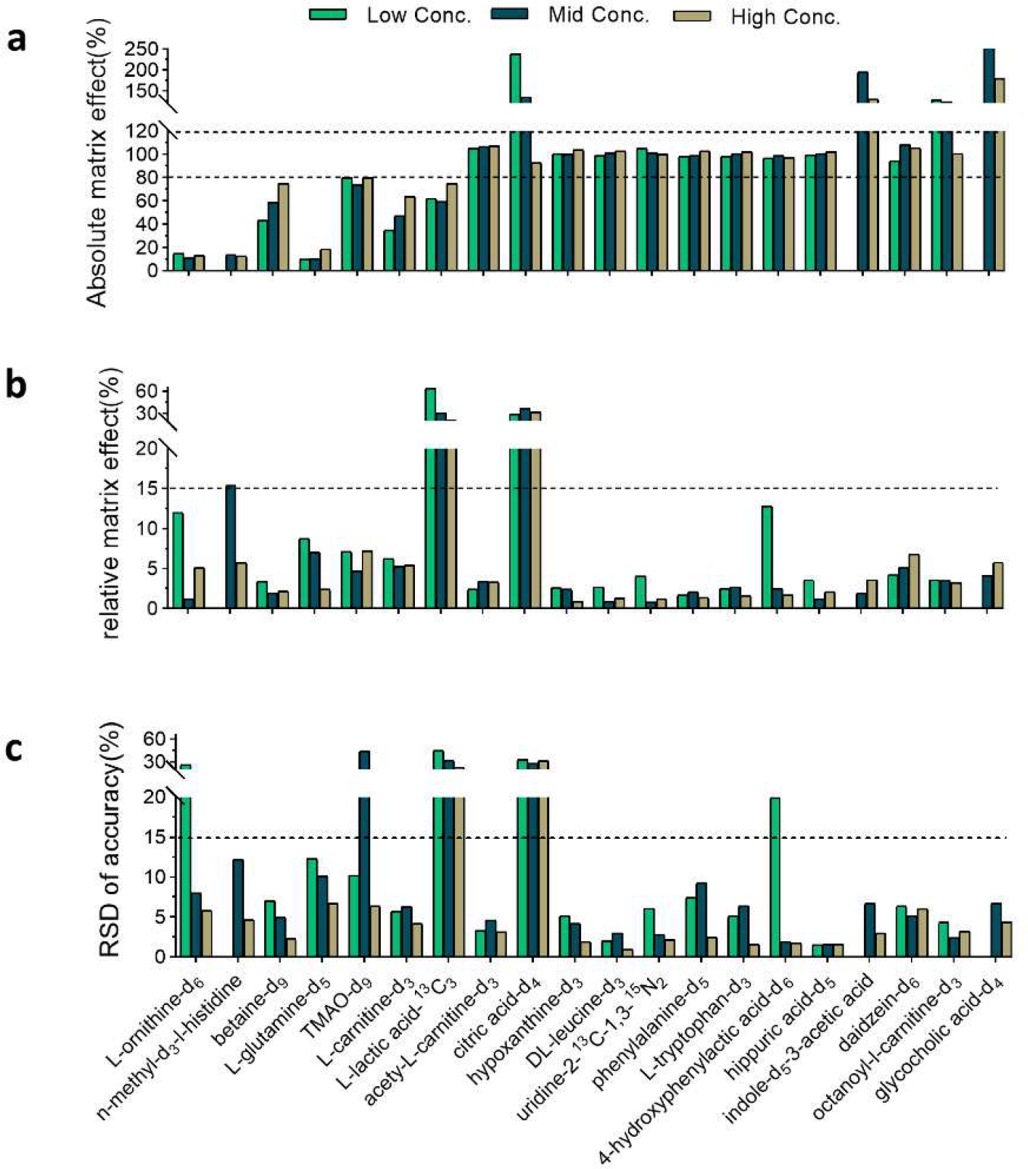
Matrix effect and precision of accuracy for spiked SIL targets in plasma. (a) Absolute matrix effect (AME). The dashed lines point out the range of 80–120%. (b) Relative matrix effect (RME). The dashed line indicates the RME at 15%. (c) Precision (RSD%) of the accuracy among four different donors. The dashed line indicates the RSD% at 15%.

The results of the matrix effect evaluation are presented in Figure 1. As shown in Figure 1a, for 45% of the SIL targets, the absolute matrix effects (AMEs) met the criteria acceptable by most bioanalytical laboratories (80%–120%)^46^ at all the concentration levels. Severe AMEs were observed for some early eluting targets (l-ornithine-*d*_6_, *n*-methyl-*d*_3_-l-histidine, and l-glutamine-*d*_5_) with values below 20%. TMAO-*d*_9_, l-carnitine-*d*_3_, betaine-*d*_9_, and lactic acid-^13^C_3_ had AMEs lower than 80%. These SIL targets eluted in regions with a high intensity of coeluting ions, as shown in the total ion chromatogram (TIC) (see Figure S6); therefore ion suppression could be expected for compounds eluting in those regions. The AMEs of citric acid-*d*_4_ and octanoyl-l-carnitine-d_3_ were above 120% at low and medium concentrations, while indole-*d*_5_-3-acetic acid and GCA-*d*_4_ had AMEs larger than 120% at all the detected concentrations. The precision of the AME was determined by the RSD of the AME, which is also called the relative matrix effect (RME). As presented in Figure 1b, l-lactic acid-^13^C_3_ and citric acid-*d*_4_ had RMEs larger than 15%, and the other targets all had RMEs less than 15%.

#### 3.1.2 Feces Validation

The linearity range and precision are summarized in Table S8. All the targets, except u-15N-guanosine, obtained good linearity (*R*^2^ > 0.98) with a wide range, and at least six consecutive calibration points were included for building the calibration curve. Good precision (RSD < 15%) was achieved for most of the calibration points (Table S8). Additionally, good accuracy (80–120%) was obtained for almost all the targets (Figure S7). Nevertheless, slightly lower accuracy was observed at either low or high concentrations for some targets (hippuric acid-*d*_5_, l-tyrosine-^13^C_9_-^15^N, dl-leucine-*d*_3_, phenylalanine-*d*_5_, and L-tryptophan-*d*_3_) because they were close to the boundary of the linear range. The accuracies of choline-*d*_4_ and dl-proline-*d*_7_ at the high level are lower than 60% due to exceeding the linear range, and the low levels of some targets were excluded because they were below the lower detection limit.

The recovery for fecal LLE extraction was validated at low and high concentration levels (Figure S8a). The RSD of recovery among four replicates was calculated to show the repeatability of the extraction process (Figure S8b). Overall, although almost all targets had a recovery below 80%, good repeatability (RSD < 10%) was obtained. However, attention needs to be paid to cytidine-^15^N_3_, u-^15^N-guanosine, and citric acid-*d*_4_, which have recoveries below 30%.

**Figure 2:**
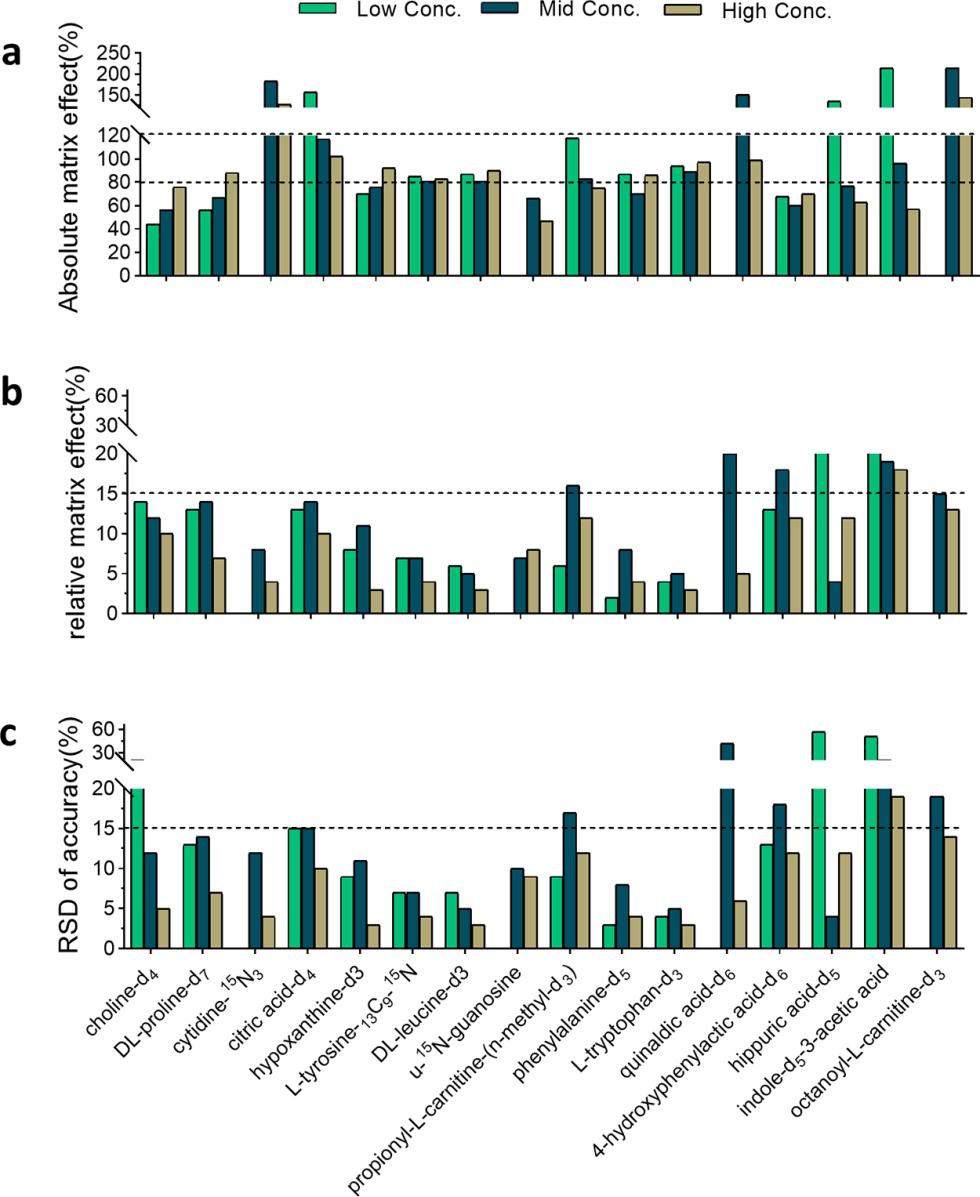
Matrix effect and precision of accuracy for spiked SIL targets in feces. (a) Absolute matrix effect (AME). The dash lines point out the range of 80–120%. (b) Relative matrix effect (RME). The dashed line indicates the RME at 15%. (c) Precision (RSD%) of the accuracy among four different donors. The dashed line indicates the RSD% at 15%.

The matrix effect results for spiked SILs in feces are described in Figure 2. Overall, the AME for most spiked SILs was around 80%, at least for two concentration levels, except cytidine-^15^N_3_ and octanoyl-l-carnitine-d3 with AMEs above 120% for all detectable concentrations (Figure 2a). The overall ion suppression for all the SILs spiked in fecal sample aligns with the intensity variation of TICs for fecal samples, as presented in Figure S6. An RME below 15% was obtained for most of the spiked SILs, with only indole-*d*_5_-3-acetic acid showing larger variability at three concentration levels (Figure 2b).

In conclusion, by validating the method with selected SILs, we explored the linear dynamic range of different classes of compounds measured in plasma and feces and also demonstrated that our method has good precision and accuracy and acceptable recovery and recovery repeatability. Additionally, the matrix effects of plasma and feces were assessed with selected SILs. In our validation, we used the original terms AME and RME to describe the matrix effect evaluation to avoid confusion. An AME value above 100% indicates ion enhancement, and that less than 100% indicates ion suppression.^31^ Although most of the bioanalytical laboratories use 80–120% as the criteria for AME,^46^ besides the acceptable RME criteria (<15%), there is no admissible value suggested by the EMA guideline. Therefore, this demonstrates that guaranteeing the reproducibility of AME is more critical for measurable compounds in bioanalytical method validation. Our validation data shows that l-lactic acid-^13^C_3_ and citric acid-*d*_4_ in plasma, and indole-*d*_5_-3-acetic acid in feces have RMEs larger than 15%. To elucidate the impact of RME on the reproducibility of quantification, the values for precision (RSD %) of accuracy for spiked SILs are plotted for plasma and feces in Figure 1c and Figure 2c, respectively. The RSD% accuracy values in both matrices align with the RME trends. The three targets with larger RME have accuracy RSD % above 15%, indicating that a high RME affects the accuracy and reproducibility of measurements among samples.

**Figure 3:**
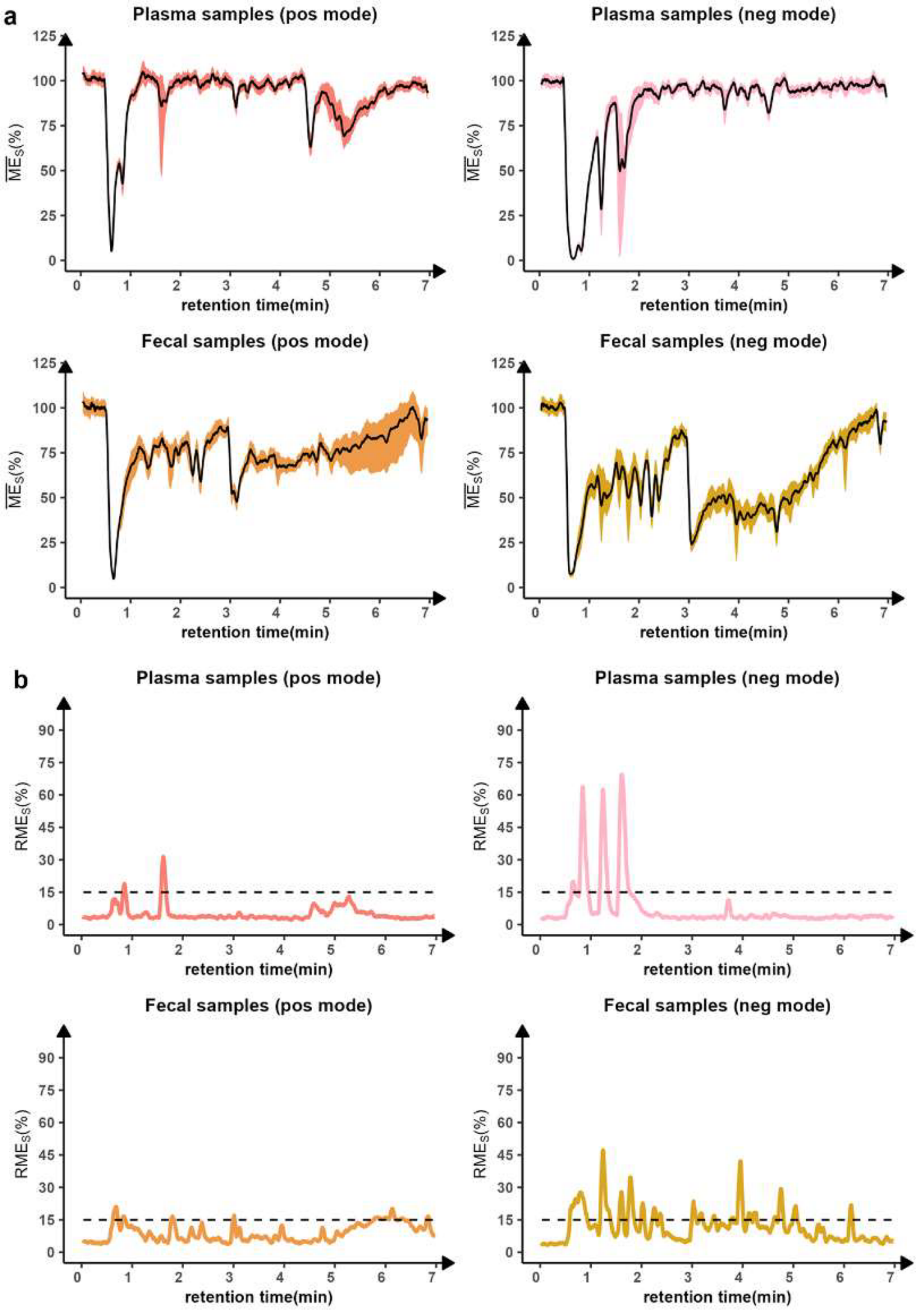
AME and RME profiles in plasma and feces. (a) AME monitoring of plasma and feces using samples from four individuals in positive and negative mode. The solid line represents the averaged absolute matrix effect profile (MEP), and the shaded area shows the MEP variations among different individuals. (b) RME monitoring in plasma and feces using samples from four individuals in positive and negative modes.

It is worth noting that, as suggested by the EMA guideline, it is possible to compensate for both AME and RME with internal standards in targeted metabolomics. In untargeted metabolomics, however, this approach is not feasible due to the unknown identity of some features and the lack of appropriate internal standards. To ensure the accuracy and reliability of data detection and interpretation, it is imperative to obtain information on the RMEs of all detected features in untargeted metabolomics measurements. With validation utilizing a wide diversity of SILs, we have highlighted the problem of the matrix effect variation in plasma and feces, while a comprehensive analysis of matrix effect variation for all detected features is still missing. Hence, how to evaluate or at least monitor the overall matrix effect variability in one or different types of matrices in untargeted metabolomics is a highly relevant problem to be addressed.

### 3.2 Matrix Effect Monitoring with PCI Compounds

In order to monitor the overall AME and RME for plasma and fecal samples, we have developed a PCI approach using xenobiotic compounds. The infusion profile of each PCI compound was acquired with different plasma samples (*n* = 4), different fecal samples (*n* = 4), and blank samples in both positive and negative ion modes. The matrix effect profile (MEP) of each sample assessed with every PCI compound was generated, and distinct MEPs were obtained for different samples with all PCI compounds (Figure S9). Those MEPs were utilized to assess the AME and RME in plasma and feces, as described in the data processing section (section 2.6).

#### 3.2.1 AME Monitoring with PCI Compounds

To ensure a fair assessment of AME and RME, a PCI compound-independent MEP was generated for each individual plasma and fecal sample. The averaged MEP intensity () was calculated for each sample to form the PCI compound-independent MEP (represented by the solid line in Figures S10–S13). The MEP variation plots with different individuals were created in both polarities accordingly, and the variation range among different individuals is represented by the shaded area in Figure 3a. Additionally, the averaged MEP intensity of the four samples was used to construct a real-time profile of the AME (represented by the solid line in Figure 3a).

The AME profile provides a qualitative evaluation of the matrix effect in plasma and feces. Ion enhancement was rare in both matrices, while ion suppression was observed in specific regions of plasma and almost the entire chromatogram of feces. Severe ion suppression occurred before 1 min regardless of matrix and polarity, likely caused by unretained nonvolatile solutes such as highly polar metabolites and ionic species (e.g., inorganic electrolytes, salts).^47,48^

In plasma, the matrix effect dropped below 60% at around 1.6 min in both polarities, at around 4.6 min in positive polarity, and at 1.2 min in negative polarity. The mass spectrum in those regions was inspected and showed a high signal of citric acid (RT at 1.58 min) and lactic acid (RT at 1.25 min) in at least one of those plasma samples (Figures S14 and S15). This suggests that citric acid and lactic acid are most likely the causes of the drastic signal decrease observed at around 1.6 and 1.2 min, as a high concentration of coeluting compounds has been considered one of the prime factors to induce ion suppression.^48^ Nevertheless, no other feature with a high signal was recorded around 4.6 min in the plasma samples, presumably due to an undetected compound or compounds outside our targeted mass range (60–800 Da).We suspect that compounds with higher masses could be suppressing the signal of small molecules we detected during this elution time.^49^ Furthermore, a high signal of EDTA was detected in plasma samples at approximately 1 min. This suggests that EDTA, a widely used anticoagulant, is a contributing factor to the significant ion suppression observed in plasma, which is consistent with reviewed literature.^50,51^ Phospholipids, a recognized source of matrix effect in plasma,^52,53^ were not observed in our study, since the lipids elute after 7 min, which is when the LC flow was diverted to waste.

Similar to plasma, lipids are also considered as one of the major sources of matrix effect in feces.^54^ However, compared to plasma, the matrix complexity of feces makes it more challenging to investigate the sources of ion suppression. We zoomed in on the mass spectrum where the most severe ion suppression occurred in feces (around 1 and 3 min) (Figure S16 and S17), but we only putatively matched the prominent signal observed at around 3 min in positive polarity with phenylalanine according to our in-house target library. Further efforts would be required to identify the coeluting compounds that induce matrix effect in feces, but this was considered beyond the scope of this study.

#### 3.2.2 RME Monitoring with PCI Compounds

The variation in the AME (shaded area in Figure 3a) shows the matrix diversity of plasma and feces between different individuals. Accordingly, the RSD% of the AME indicates the RME of the entire runs (Figure 3b). In the positive ion mode, the monitored RME in plasma and feces remains around or below 15% throughout almost the entire chromatogram. However, around 1.6 min in plasma, the RME exceeds over 30%, which is likely due to a large concentration variability of citric acid in those samples. In the negative ion mode, there are more regions with high monitored RME in both plasma and feces. Three major spikes in the RME plot, up to 60%, are observed in plasma, and two of them are probably caused by high concentration variability of lactic acid and citric acid. In feces, the RME fluctuates within 45% in most regions. The RME overview demonstrates that it is reasonable to compare the detected signals in plasma or feces from different donors across most regions of the chromatogram, regardless of severe AME. Still, caution should be exercised for certain regions, particularly in the negative ion mode.

**Figure 4:**
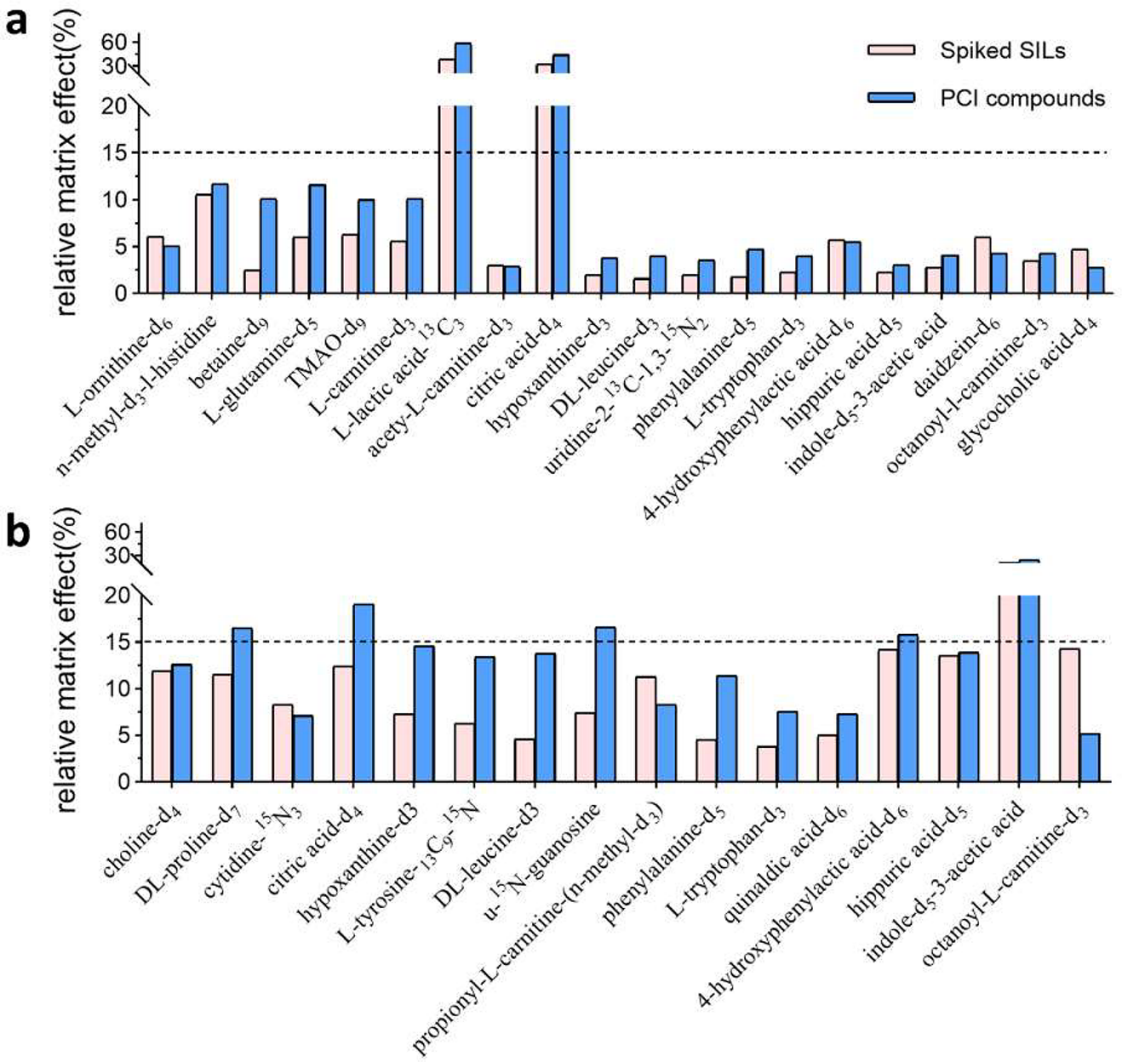
Comparison of the RME evaluated with spiked SILs and PCI compounds in (a) plasma and (b) feces. The averaged RME data from different concentrations of the spiked SILs were used. For the SILs that are detectable in both polarities, the selected polarity is consistent between the two methods.

To validate the accuracy of using PCI compounds to monitor the RME, we extracted the monitored RME values at specific time points matching the RT of the spiked SILs and compared them to the RME assessed with spiked SILs (Figure 4). The results reveal consistency between the RME monitored by PCI compounds and the RME assessed using spiked SILs. In plasma, both evaluation methods demonstrated that l-lactic acid-^13^C_3_ and citric acid-*d*_4_ had an RSD% around 30%, while the other SILs had acceptable RSD% values (<15%). In feces, both methods indicated that indole-*d*_5_-3-acetic acid had high variability (RSD > 15%). These results demonstrate that using PCI compounds for RME evaluation is comparable to spiking SILs, making it a compelling approach to evaluate RME for both known targets and unknown features.

### 3.3 RME Monitoring Application to Targets Included in an In-House LC-MS Library

**Figure 5:**
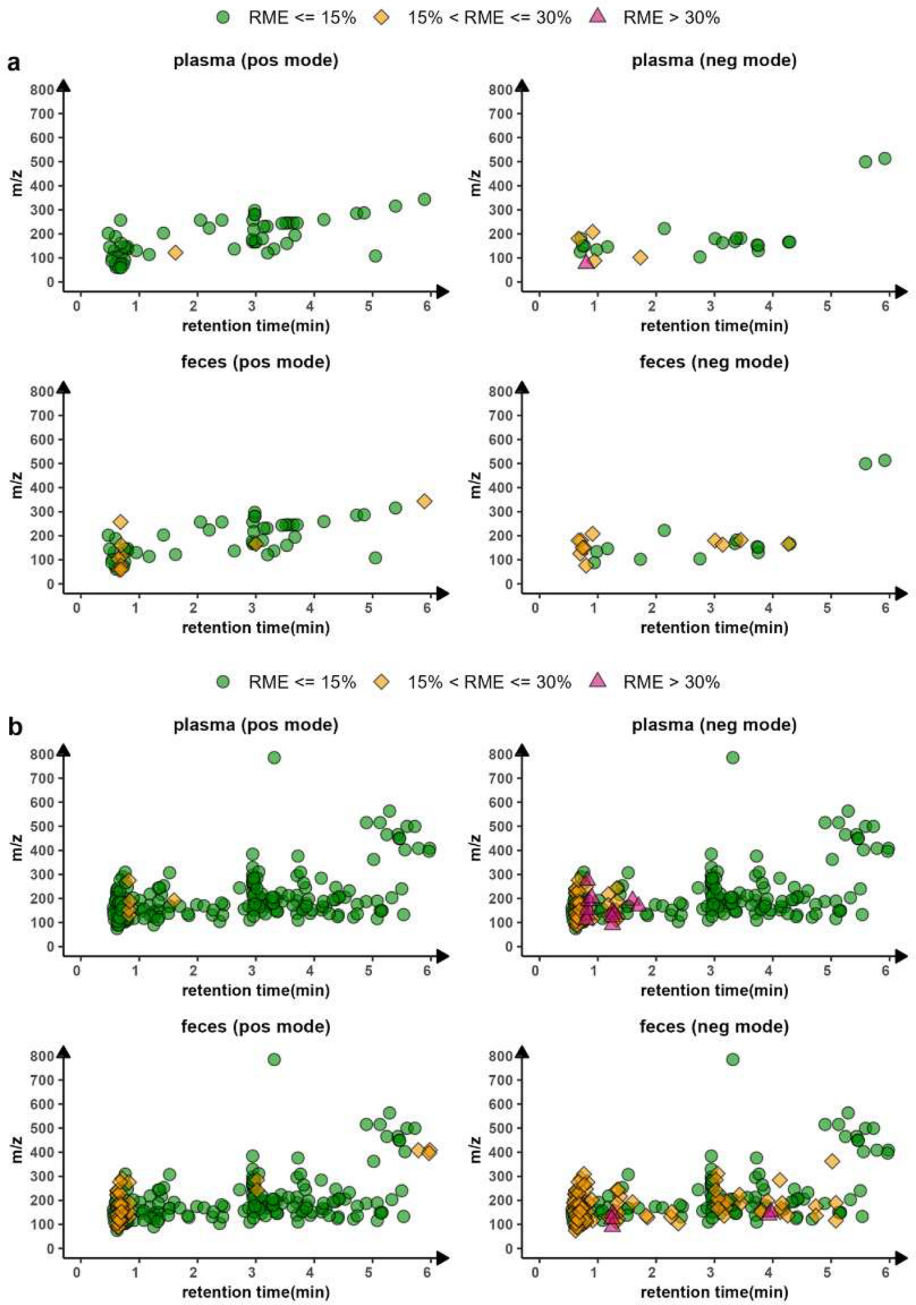
RME assessment of targets included in the in-house library. (a) Predicted RME by PCI compounds for targets that are only detectable in one polarity mode: positive (55 targets) or negative (25 targets). (b) Predicted RME by PCI compounds for targets that are detectable in both positive and negative modes (225 targets).

Together with the LC-MS untargeted method, an in-house targeted library containing retention time and accurate mass information was established by measuring commercially available authentic standards. The library included 305 targets that eluted before 6 min, and those targets were distributed across various classes, including amines, benzenoids, organic acids, indoles, nucleosides, nucleotides, and bile acids. In light of the effectiveness of PCI compounds on RME monitoring, we predicted the RMEs of the 305 targets based on their RT and the acquired RME profiles in plasma and fecal samples, respectively. Figure 5a provides an overview of the predicted RMEs for 55 targets that are only measurable in the positive ion mode and 25 targets that are only detectable in the negative ion mode (refer to Table S9 for more information about the targets and predicted RME values). As expected, there were more targets within a caution zone (15% < RME ≤ 30%) in feces than in plasma. A higher proportion of targets in the negative ion mode were predicted to be affected by sample diversity compared to positive ion mode. In plasma, only one target (glycolic acid, with an RT of 0.80 min) detected in the negative ion mode shows RSD > 30%. Figure 5b presents the predicted RMEs of the 225 targets that are detectable in both positive and negative modes (refer to Table S10 for more information about the targets and predicted RME values). In general, we observed that more targets are susceptible to the matrix effect diversity in the negative ion mode than in the positive ion mode, regardless of matrix type, and that there are more targets predicted with a RME > 15% in feces compared to plasma. For the targets that are detectable in both ionization modes, the predicted RME needs to be taken into account when selecting the appropriate polarity for quantitation, along with other parameters such as signal-to-noise ratio.

Although the predicted RME in our study is only based on four individual plasma and fecal samples and only the predicted value at the apex of the peak was used (without considering the peak width), our results demonstrate the potential of the PCI approach in identifying the regions of caution regarding RME and predicting RME for both known and unknown features based on their retention times. Some high-resolution MS instruments have the option to continuously infuse a compound after LC separation for calibration purposes, which also can be utilized for ME monitoring. However, including multiple PCI compounds enhances the possibility of capturing various ME profiles compared to using just one, as demonstrated in our study, especially for fecal samples in negative mode (Figure S9d). Moreover, ideal PCI compounds should have exogenous *m*/*z* values that do not interfere with the targets of interest and should not induce significant additional ME. Overall, we strongly recommend applying a PCI approach both during the method development and routine studies. Its application in method development aids in identifying cautionary areas in the chromatography that suffer from the matrix effect. This information is crucial in guiding the optimization of specific LC parameters, such as gradient and injection amount, to minimize matrix effect. Additionally, the routine application of PCI is crucial in improving the reliability of data interpretation in studies that apply untargeted methods, particularly for cohorts with an anticipated range of abnormal or unusually high compound concentrations. For instance, plasma samples from individuals with kidney disease may exhibit wider zones of ion suppression due to the specific nature of the health condition, which involves the accumulation of various compounds in the blood. Likewise, when comparing fecal samples from individuals consuming a ketogenic diet with those from vegetarians, it is important to examine ion suppression due to the high variation in fat content.

## 4 Conclusion

In this study, we propose a comprehensive framework for the development of untargeted metabolomics methods with a PCI approach for matrix effect monitoring. To the best of our knowledge, our research is the first study offering practical strategies that combine the optimization of the sample injection amount and the reconstitution solvent, performance validation, and matrix effect evaluation in the development of an untargeted metabolomics method.

Our study demonstrates that optimization of the sample injection amount, utilizing ion transmission monitoring techniques such as ITC in the TripleTOF system, is critical for balancing metabolite coverage and signal linearity. Additionally, considering specific LC gradients and metabolite classes of interest, it is crucial to optimize reconstitution solvents to avoid potential issues of peak shape distortion and poor solubility in untargeted methods. Furthermore, validating an untargeted metabolomics method in a targeted manner provides valuable insights into the analytical performance of the method, including the linear dynamic range, precision, accuracy, recovery, and matrix effect.

To address the challenge of matrix effect, we highly recommend implementing a PCI approach during the development phase of an untargeted metabolomics method and suggest also applying it in routine studies. Our results demonstrate that the PCI approach effectively monitors the matrix effect for plasma and fecal samples, allowing the identification of regions with high matrix effect variation in the untargeted metabolomics method that should be interpreted with caution. More impressively, the PCI approach yields comparable RME data when compared to the traditional postextraction spiking method, making it a compelling technique for assessing RMEs for both known targets and unknown features detected in untargeted metabolomics. This approach shows great promise for generating reliable data from an untargeted method and advancing quantitative analysis in untargeted metabolomics.

## Supporting Information Available

General experimental information, calibration lines, final concentration of each PCIS compound, dilution ratio calculation, validation parameters, and predicted RMEs (XLSX)
Additional method optimization and experimental details, overlapped MEPs, and mass spectra (PDF)
