# Supplementary material for: Development of an Untargeted LC-MS Metabolomics Method with Postcolumn Infusion for Matrix Effect Monitoring in Plasma and Feces
Source: J Am Soc Mass Spectrom. 2024 Feb 21;35(3):590–602. doi: 10.1021/jasms.3c00418 (PMC10921459; doi:10.1021/jasms.3c00418)
Supplement: Supplementary file 2 — js3c00418_si_002.pdf [file js3c00418_si_002.pdf]

Supporting information

Development of an untargeted LC-MS metabolomics method with post-column infusion for matrix effect monitoring in plasma and feces

Pingping Zhu[1], Anne-Charlotte Dubbelman[1,2], Christie Hunter[3], Michele Genangeli[1], Naama Karu[1†], Amy Harms[1], Thomas Hankemeier[1*]

[1]Metabolomics and Analytics Centre, Leiden Academic Centre for Drug Research, Leiden University, Leiden 2333 CC, Netherlands

[2]Institute for Risk Assessment Sciences, Utrecht University, Utrecht 3584 CM, the Netherlands

[3]SCIEX, Redwood City, CA 94065, USA

[†]Current address: TIMACS, Hobart, Tasmania 7000, Australia.

[*]Corresponding author: Prof. Thomas Hankemeier, Metabolomics and Analytics Centre, Leiden Academic Centre for Drug Research, Leiden University, Leiden 2333 CC, Netherlands

E-mail: hankemeier@lacdr.leidenuniv.nl , Tel: +31-715274226

Table of Contents

Table of Contents S2

Method optimization: reconstitution solvent and injection amount S3

Figure S1. Distribution of endogenous compound abundance for selected metabolites. S5

Figure S2. Reconstitution solvent comparison in plasma and feces. S6

Figure S3. Injection concentration optimization for plasma and feces. S7

Figure S4. Matrix effect profile and ion transmission control (ITC) plot for all the injection combinations of plasma in positive mode S7

Figure S5. Recovery (a) and accuracy (b) of the spiked SILs in plasma S8

Figure S6. Averaged AME of spiked SILs and TIC intensity in plasma and feces. S8

Figure S7. Accuracy of spiked SILs in feces S9

Figure S8. Recovery (a) and RSD of recovery (b) of the spiked SILs in feces S9

Figure S9. Matrix effect profile (MEP) of each sample with all the PCI compounds S10

Figure S10. Overlapped MEPs of four PCI compounds for each plasma sample in positive mode. S10

Figure S11. Overlapped MEPs of four PCI compounds for each plasma sample in negative mode. S11

Figure S12. Overlapped MEPs of four PCI compounds for each fecal sample in positive mode. S11

Figure S13. Overlapped MEPs of four PCI compounds for each fecal sample in negative mode. S12

Figure S14. Mass spectrum inspection of the suppressed areas in plasma samples with 5-fluoroisatin in positive mode S13

Figure S15.Mass spectrum inspection of the suppressed areas in plasma samples with fludrocortisone in negative mode S14

Figure S16. Mass spectrum inspection of the suppressed areas in fecal samples with 5-fluoroisatin in positive mode S15

Figure S17.Mass spectrum inspection of the suppressed areas in fecal samples with fludrocortisone in negative mode S16

Method optimization: reconstitution solvent and injection amount

In developing the RPLC-MS untargeted method, we first optimized the reconstitution solvent for feces and plasma by reconstituting dried extracted samples in 100% water, water/ACN (9:1, v/v), and water/ACN (8:2, v/v). The chromatographic peak shape and height of representative metabolites were evaluated for reconstitution solvent selection.

Figure S2a presents the chromatography of the early eluting endogenous metabolite guanine and the late eluter deoxycholic acid (DCA) in both plasma and fecal samples with three reconstitution solvents. Peak shape deterioration of guanine was observed with the increased ACN in the injection solvent. It has been reported that peak distortion of polar metabolites in RPLC is caused by viscosity and elution strength mismatch between the injection solvent and the mobile phase, and that a potent injection solvent causes peak splitting and fronting due to the migration time differences between the analyte, injected solvent and mobile phase[1–3]. In our gradient, the proportion of mobile phase B reaches 20% around 2.5 min, at which the strength and viscosity of the mobile phase is equal to the injection solvent. This explains why peak distortion only occurred for metabolites eluting before 2.5 min, like guanine, when injected with 20% ACN in our method, and the peak shape of metabolites eluting after 2.5 min, like DCA, were retained.

Interestingly, although the peak shape of DCA was not distorted, the peak height increased along with the increment of ACN in both feces and plasma. We also observed that the signal boost of DCA caused by ACN is much higher in feces than in plasma. This phenomenon suggests that adding ACN to the reconstitution solvent facilitates the solubility of DCA regardless of sample type. However, the signal improvement is sample or concentration related, as the concentration of DCA in feces is higher than in plasma.

Lindahl *et al.* reported that when comparing reconstitution with different proportions of MeOH in water, using 100% water as a reconstitution solvent increases the response of metabolites with $\log P < 5$ [4]. However, the data we obtained about DCA ($\log P = 3.5$)[5] disagrees with their conclusion and restricts the range to lower logP. To investigate the correlation of metabolite polarity and the effect of reconstitution solvent on metabolite response in our method, we compared the peak areas of 26 endogenous metabolites in feces with three injection solvents (Figure S2b). These 26 metabolites were widely distributed in retention time (RT), representing a wide range of polarity. As shown in Figure 1b, the peak areas were comparable in three reconstitution solvents for the metabolites eluting before 6 min, while after that, the peak areas increased (area percentage higher than 33%) with raised ACN ratio in the reconstitution solvents. Together with other studies[2,4], it proves that the effect of the injection solvents on the peak shape of polar metabolites and on the solubility of less polar metabolites is ubiquitous in the RPLC method, and our result suggests that the affected regions are dependent on the injection solvent and the LC gradient. Thus, it is necessary to consider the peak shape and solubility of metabolites of interest when selecting the injection solvent for RPLC untargeted methods. We decided to use 100% water with 0.1% FA as the final injection solvent as we are more interested in polar and semi-polar metabolites. For metabolites eluting after 6 min, more caution is needed in interpreting the results in clinical applications, given their potentially incomplete solubility. This aspect was not further examined in plasma, as the peak distortion and solubility are more dependent on the LC method and metabolites rather than the sample itself.

Next, we investigated the effect of the dilution factor (DF) and injection volume on metabolome coverage and signal saturation for plasma and fecal samples. In total, we compared three DFs (1:6, 1:3, 1:2) in plasma (v/v) and two DFs (1:6, 1:3) in feces (mg/v), along with two injection volumes (1μL and 2μL) in both matrices. The combination of DF and injection volume is presented as DF_μL.

To compare the metabolome coverage, we integrated 47 identified metabolites (details provided in Table S6) with diverse endogenous abundance, for all the DF and injection volume combinations. Figure S3a and Figure S3b present the metabolite distributions of different combinations in plasma and feces, respectively. The 47 metabolites are detectable in all the combinations, and as expected, the overall signal increased with a higher injected concentration in both matrices. Potential signal saturation was evaluated by visualizing the count conversion factors (ccf) plots for each combination (Figure 3c-f). The %ccf value reflects the degree to which the dynamic ion transmission control (ITC) has modulated the MS ion current in TOF MS spectrum for that point in the chromatogram. This dynamic ITC is a feature of the TripleTOF 6600 system which functions to reduce the risk of detector saturation when high amounts of MS ion current are present in a particular sample. As ion current reaches a predefined upper TargetTIC (total ion chromatogram) signal, the ion current is modulated down by adjusting a lens voltage in the front end of the instrument. This adjustment is done scan-by-scan based on feedback from the detection system, the higher the ion current goes above the TargetTIC, the higher the modulation applied. The %ccf value shows the % of ion beam modulation that was used in each spectrum. ITC considers both the total ion current and the ion current of a dominant ion. Although the peak area written to the datafile is corrected back to the value it would have been at 100% ion current, ideally, to ensure quantitative linearity, an ion load that is causing maximal ion modulation or exceeding the ion modulation limit (2%) should be avoided.

The %ccf plots of injections in plasma show that, in general, the ion current is modulated to a greater extent with a higher injected concentration (Figure S3c). It reaches the lowest value around 0.85 min, where except DF6_1μL, the other combinations either surpass (DF2_2μL, DF2_1μL, DF3_2μL) or stay near (DF3_1μL, DF6_2μL) the limit of the ion modulation (Figure S3d). In fecal injections, the %ccf plots show that the ion modulation reaches the limit around 4.7 min (Figure S3f). DF6_2μL and DF3_2μL reach the limit of ion modulation, and DF3_1μL stays close to the limit (Figure S3e).

Additionally, to assess the relationship between injection amount and matrix effect, post-column infusion (PCI) compounds were used to monitor the matrix effect for all the injection combinations in plasma. As expected, a higher-injected sample amount resulted in greater ion suppression in the area that suffers from matrix effect. DF2_2 μL caused the most pronounced ion suppression and DF6_1 μL experienced the least ion suppression (Figure S4a-b). The % ccf plots exhibited similar fluctuation trends to the matrix effect profiles in those samples (Figure S4c-d), indicating that both reflect the amount of ion current injected into the MS system. When more ions are injected, there is increased ionization competition in the source, and the ITC undergoes greater modulation to reduce the risk of detector saturation.

According to the selected 47 metabolites, there is no metabolite coverage difference among all the combinations. Hence, DF6_1μL is the optimum injection condition for both plasma and feces to avoid potential signal saturation and reduce matrix effect throughout the chromatogram. However, in plasma there are more metabolites with low abundance, and DF6_1μL results in higher number of peaks with area below 1000 (Figure S3a), which may cause repeatability and quantification issues for these metabolites. Therefore, to achieve a compromise between the

signal sensitivity, detector saturation and matrix effect, DF3_1µL and DF6_1µL should be selected as the final injection condition for plasma and feces, respectively. Nevertheless, considering the limited fecal sample availability in some clinical studies, DF8_1µL was utilized for further method validation in feces. The assessment of metabolome coverage was not extended with DF8_1µL, as we do not anticipate significant signal reduction with the injection volume fold changes less than 1.5 times.

Reference:

1. Castells CB, Castells RC. Peak distortion in reversed-phase liquid chromatography as a consequence of viscosity differences between sample solvent and mobile phase. *J Chromatogr A*. 1998;805:55-61. doi:10.1016/s0021-9673(98)00042-9

2. Keunchkarian S, Reta M, Romero L, Castells C. Effect of sample solvent on the chromatographic peak shape of analytes eluted under reversed-phase liquid chromatogaphic conditions. *J Chromatogr A*. 2006;1119(1-2):20-28. doi:10.1016/J.CHROMA.2006.02.006

3. Vanmiddlesworth BJ, Dorsey JG. Quantifying injection solvent effects in reversed-phase liquid chromatography. *J Chromatogr A*. 2012;1236:77-89. doi:10.1016/j.chroma.2012.02.075

4. Lindahl A, Sääf S, Lehtiö J, Nordström A. Tuning Metabolome Coverage in Reversed Phase LC-MS Metabolomics of MeOH Extracted Samples Using the Reconstitution Solvent Composition. *Anal Chem*. 2017;89(14):7356-7364. doi:10.1021/acs.analchem.7b00475

5. Roda A, Minutello A, Angellotti MA, Fini A. Bile acid structure-activity relationship: Evaluation of bile acid lipophilicity using 1-octanol/water partition coefficient and reverse phase HPLC. *J Lipid Res*. 1990;31(8):1433-1443. doi:10.1016/s0022-2275(20)42614-8

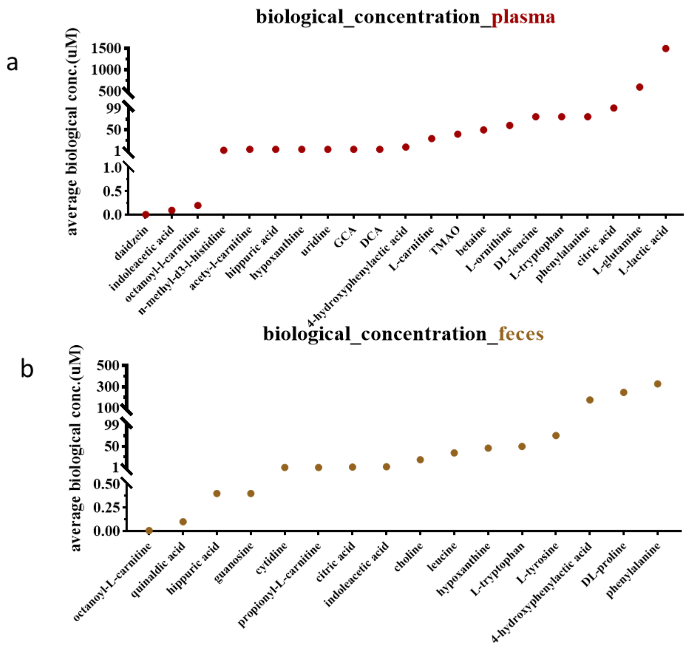

Figure S1. Distribution of endogenous compound abundance for selected metabolites. (a) metabolites selected for plasma. Abundance levels were determined by referring to the reported concentration in HMDB (b) metabolites selected for feces. Abundance levels were determined by standard addition to the collected fecal samples due to the inconsistency of the reported concentration.

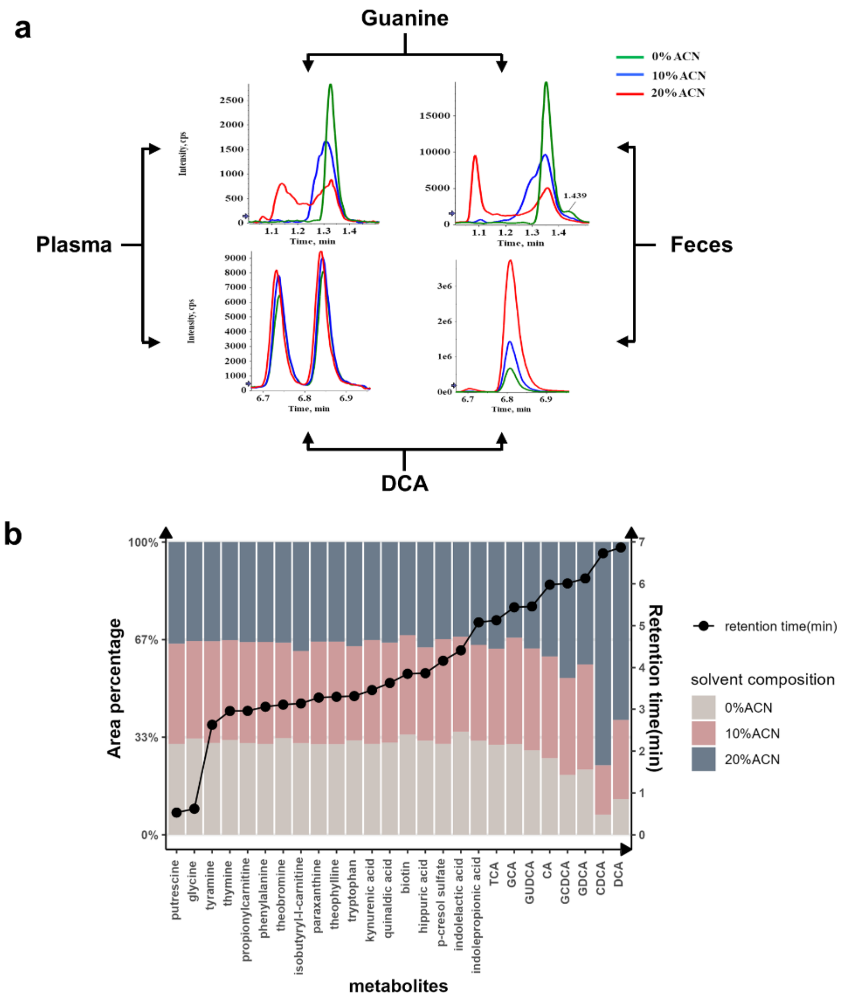

Figure S2. Reconstitution solvent comparison in plasma and feces. Plasma (dilution factor (DF) 2) and feces (DF6) were injected with 1μL in three reconstitution solvents. (a) Chromatogram of guanine and DCA. Guanine as an example of how reconstitution solvent affects the peak shape for polar metabolites; DCA (RT at 6.83min, the other peak at around 6.73 is chenodeoxycholic acid) as an example of how reconstitution solvent affects the signal for less polar metabolites. (b) Peak area comparison of 26 metabolites (bile acids were analyzed in negative mode) in feces injected with three reconstitution solvents. The peak area percentage for each metabolite was calculated by dividing the peak area in a specific reconstitution solvent by the sum of the peak area in three reconstitution solvents. If there is around 33% area percentage for each condition, no obvious solubility issue was observed for that metabolite. To avoid the impact of peak shape distortion on integration, only metabolites with acceptable peak shape in 20% ACN were involved in the comparison (metabolites which eluted around void volume or after 2.5 min). Complete names for the abbreviation: taurocholic acid (TCA), glycocholic acid (GCA), cholic acid (CA), glycochenodeoxycholic acid (GCDCA), glycodeoxycholic acid (GDCA), chenodeoxycholic acid (CDCA).

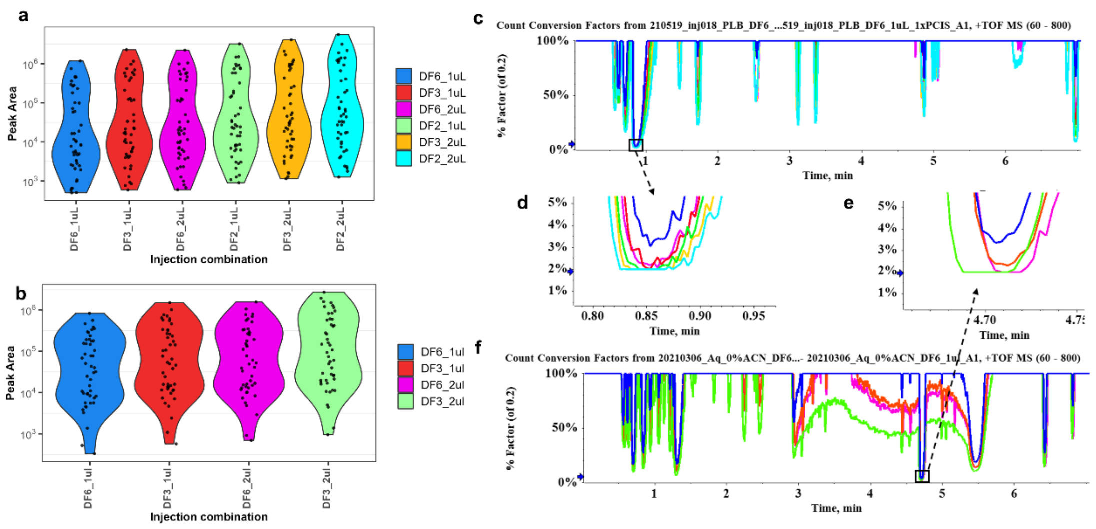

Figure S3. Injection concentration optimization for plasma and feces. Plasma and feces were reconstituted in 0.1% FA in water. In the metabolite coverage comparison, 47 metabolites (measured in positive ion mode), all eluting before 6 min to avoid the solubility issue of late-eluting compounds, were included. (a) Metabolite coverage comparison of different injected concentrations in plasma. (b) Metabolite coverage comparison of different injected concentrations in feces. (c) %Ccf plots for different injected concentrations in plasma. (d) Zoom in on the %ccf plots in the region of highest ion current modulation in plasma. (e) Zoom in on the %ccf plots in the region of highest ion current modulation in feces. (f) %Ccf plots for different injected concentrations in feces.

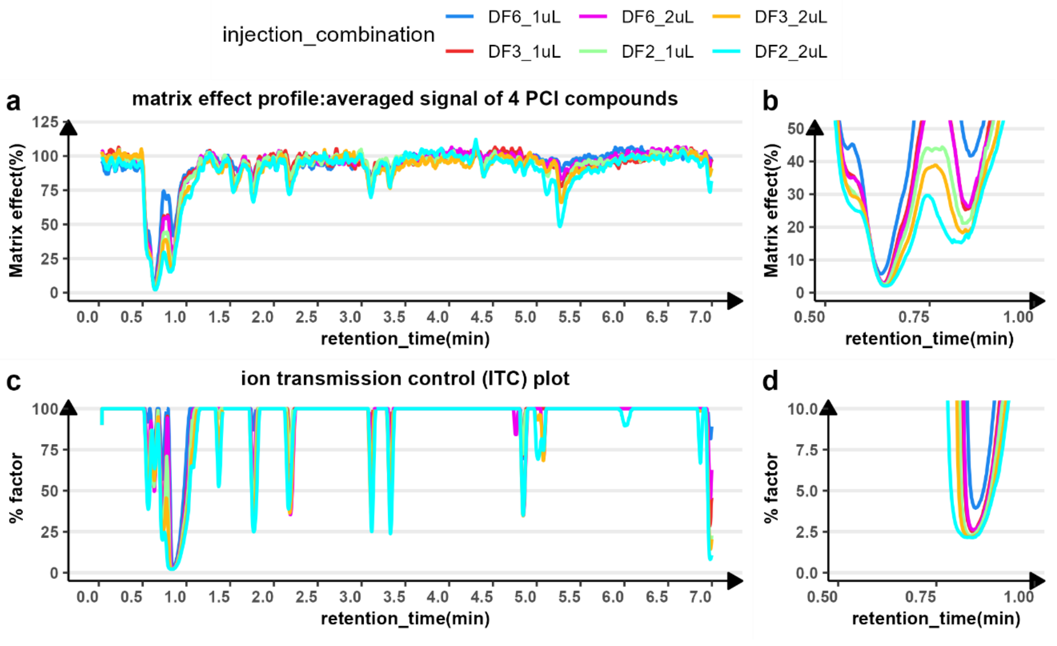

Figure S4. Matrix effect profile and ion transmission control (ITC) plot for all the injection combinations of plasma in positive mode.(a) matrix effect profile presented with the averaged signal of 4 PCI; (b) Zoom in on the plot of (a) in the region of highest ion suppression ; (c) ITC plots of all the injection combination;(d) Zoom in on the plot of (c) in the region of highest ion current modulation.

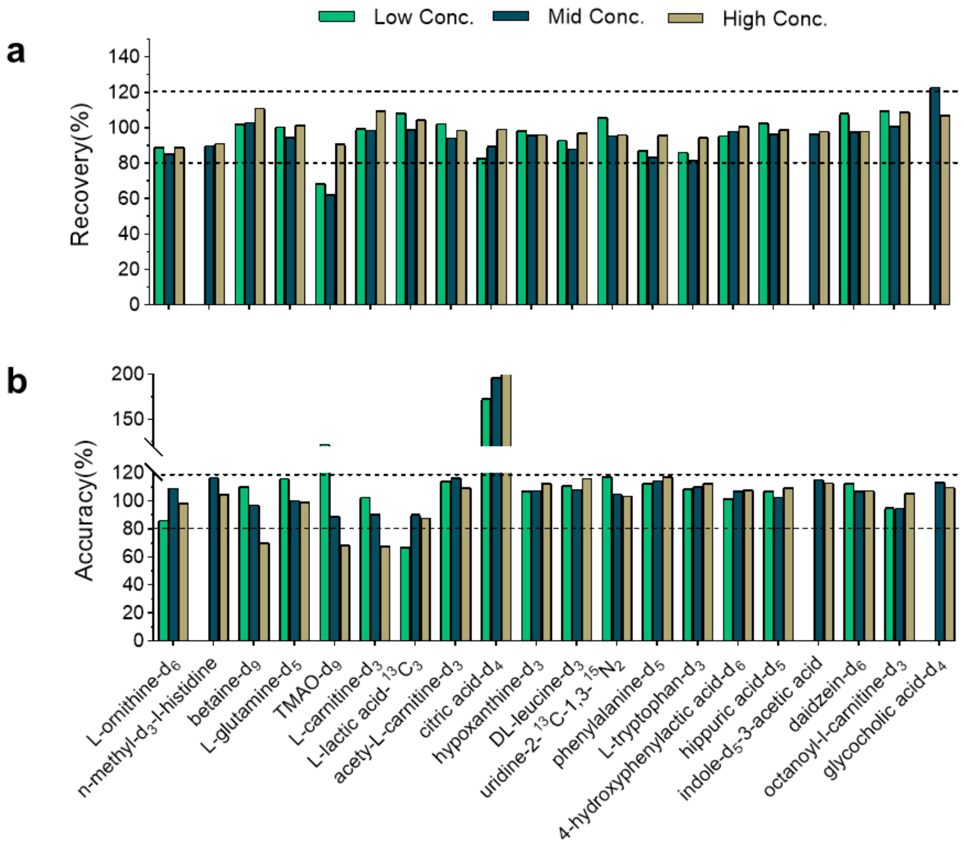

Figure S5. Recovery (a) and accuracy (b) of the spiked SILs in plasma

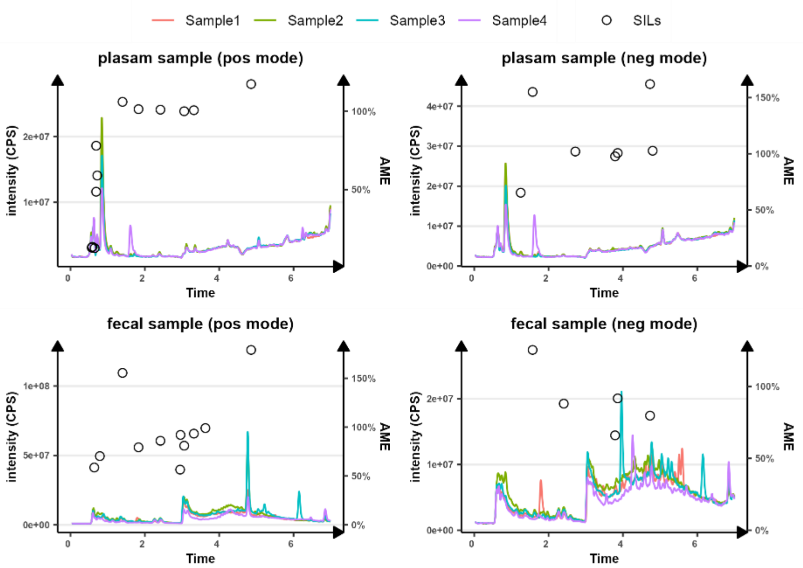

Figure S6. Averaged AME of spiked SILs and TIC intensity in plasma and feces. The averaged AME was calculated from different concentrations of spiked SILs.

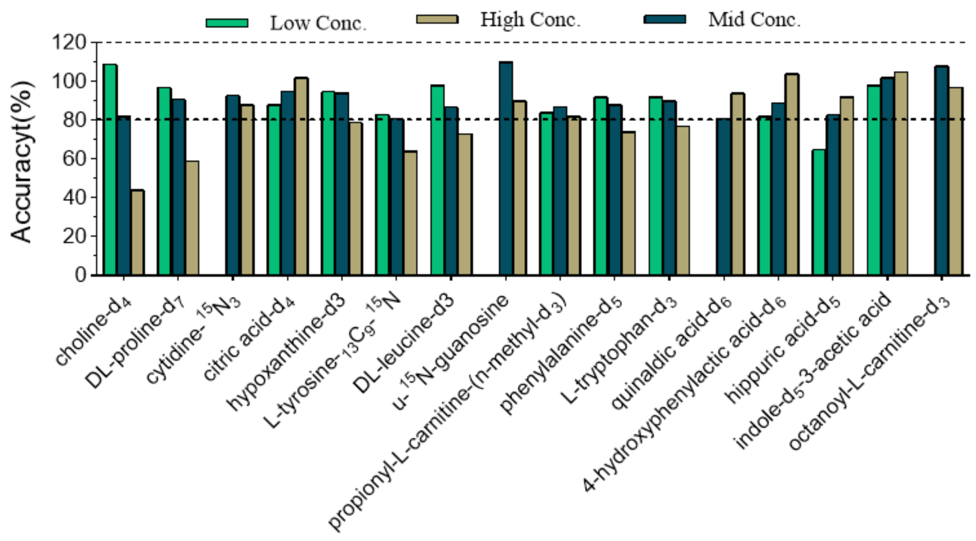

Figure S7. Accuracy of spiked SILs in feces

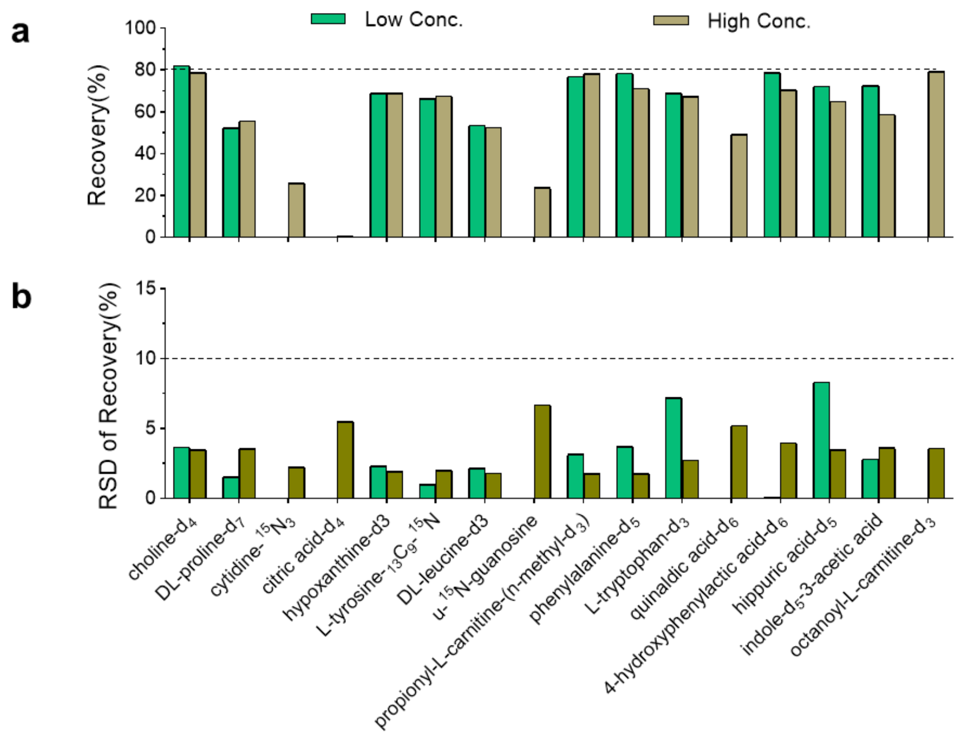

Figure S8. Recovery (a) and RSD of recovery (b) of the spiked SILs in feces

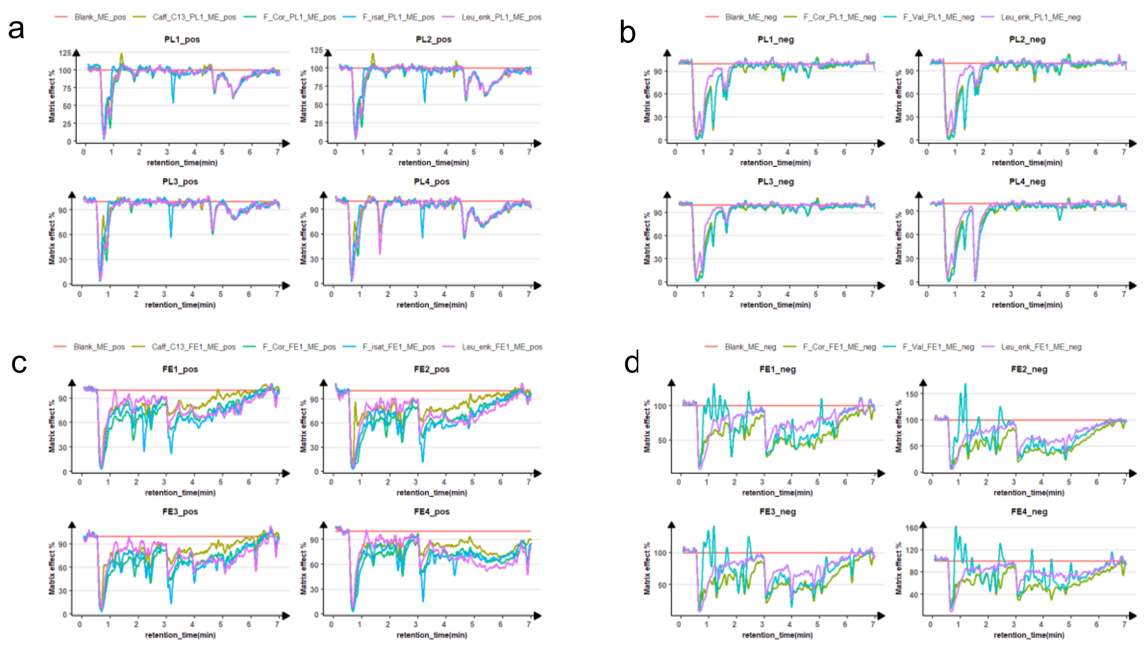

Figure S9. Matrix effect profile (MEP) of each sample with all the PCI compounds. (a) plasma in positive mode. (b) plasma in negative mode. (c) feces in positive mode. (d) feces in negative mode.

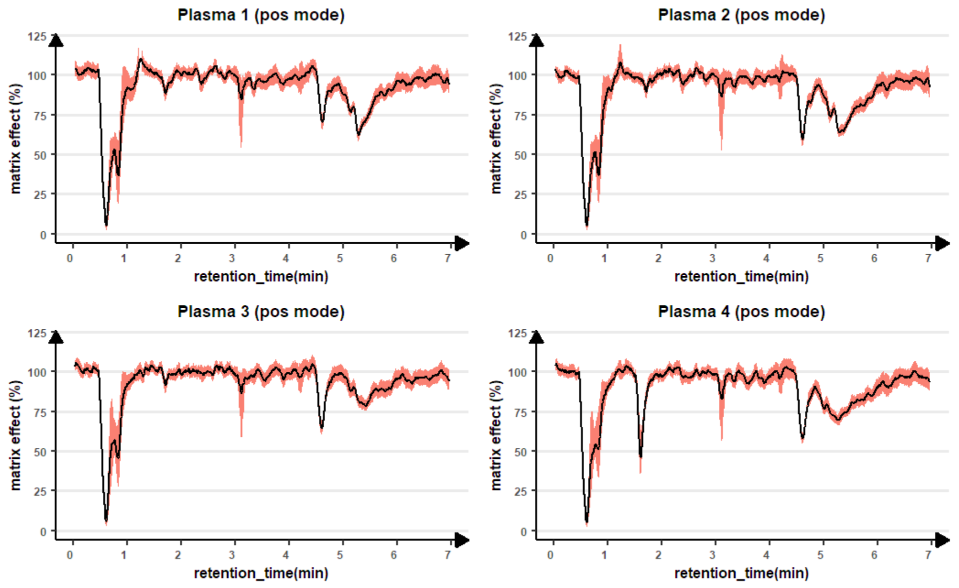

Figure S10. Overlapped MEPs of four PCI compounds for each plasma sample in positive mode.

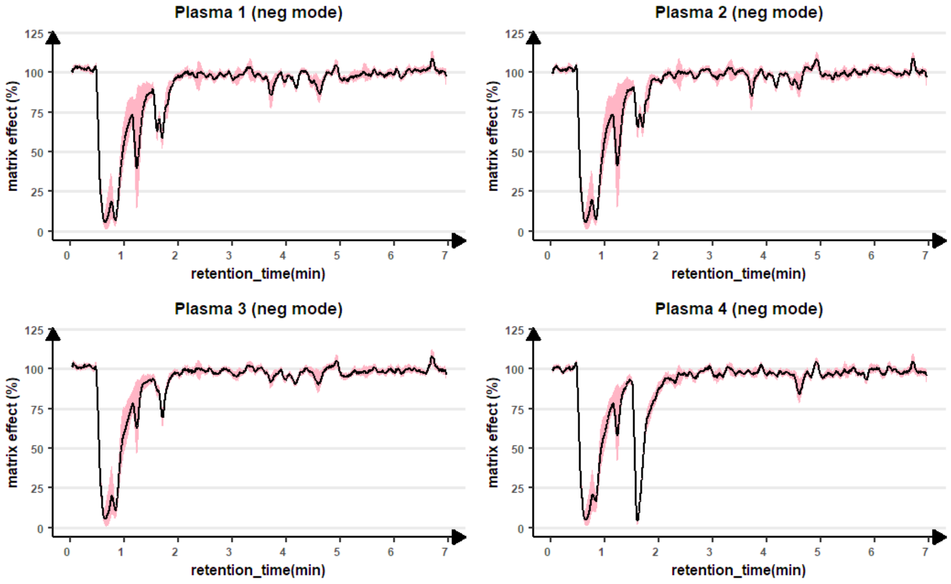

Figure S11. Overlapped MEPs of four PCI compounds for each plasma sample in negative mode.

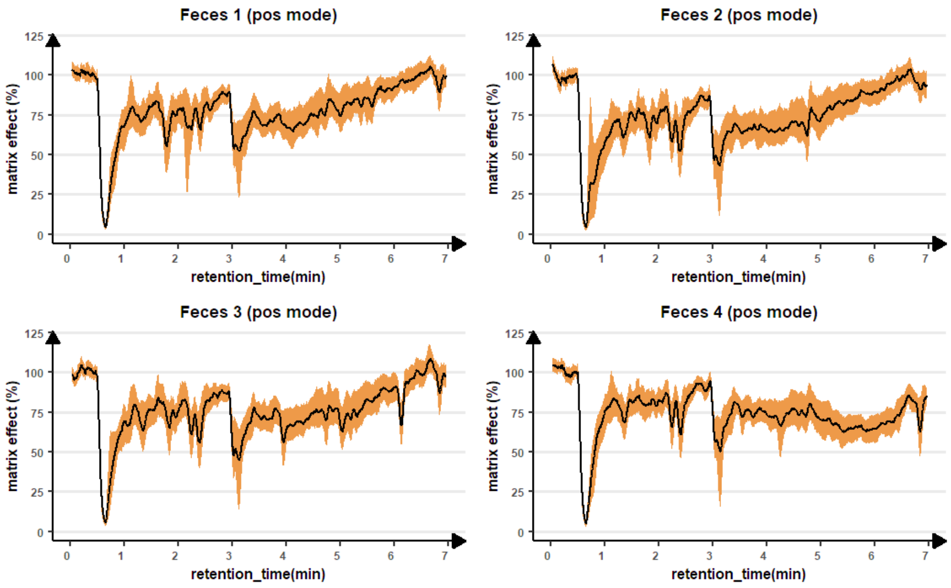

Figure S12. Overlapped MEPs of four PCI compounds for each fecal sample in positive mode.

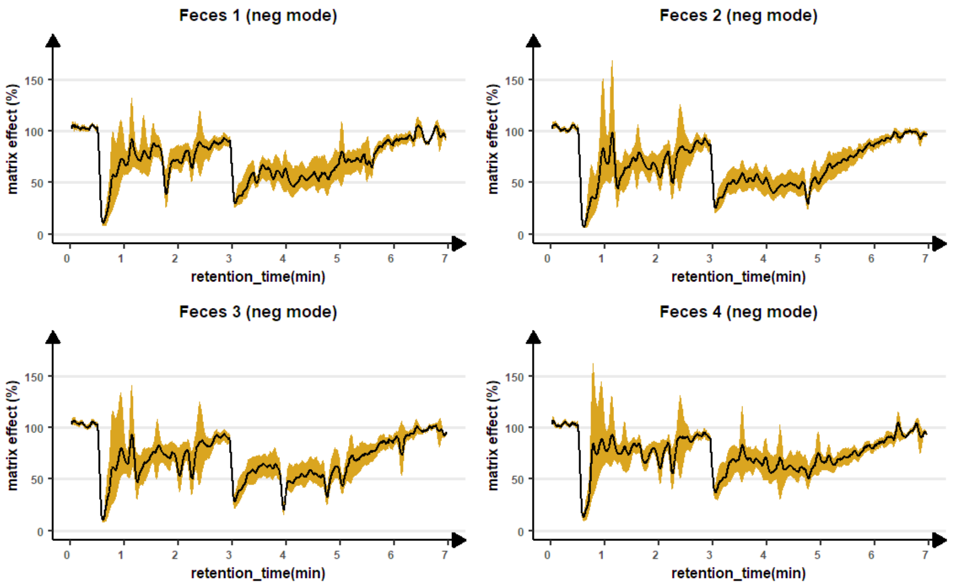

Figure S13. Overlapped MEPs of four PCI compounds for each fecal sample in negative mode.

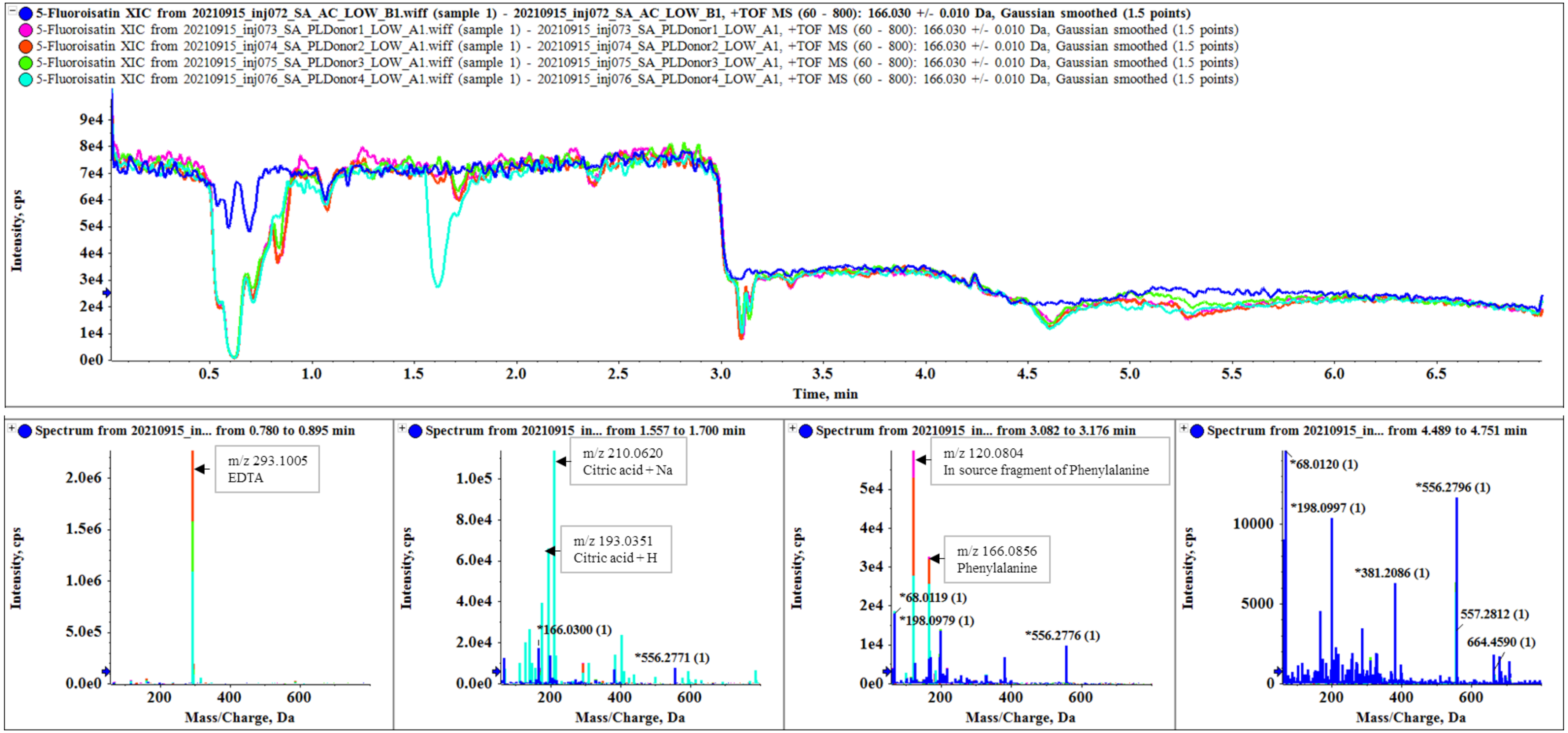

Figure S14. Mass spectrum inspection of the suppressed areas in plasma samples with 5-fluoroisatin in positive mode

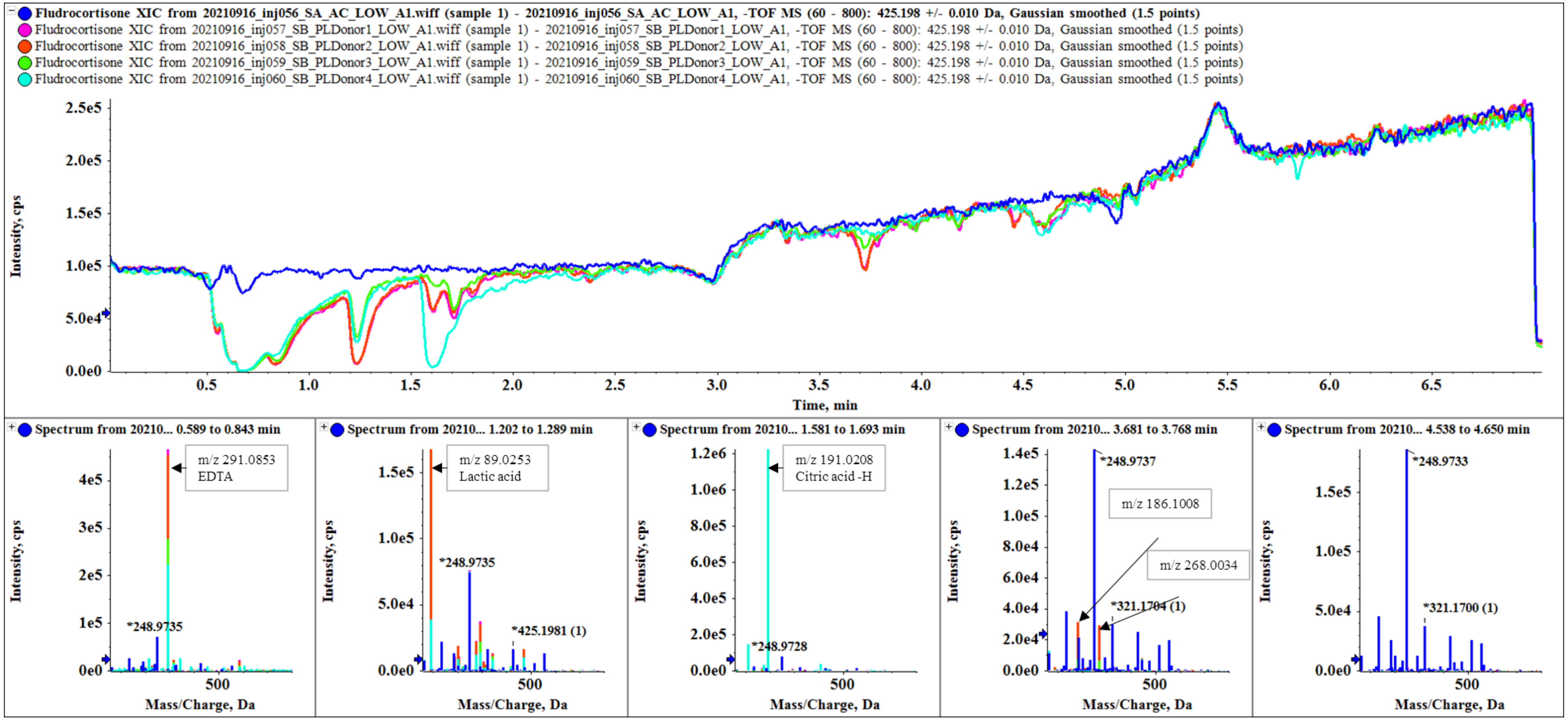

Figure S15.Mass spectrum inspection of the suppressed areas in plasma samples with fludrocortisone in negative mode

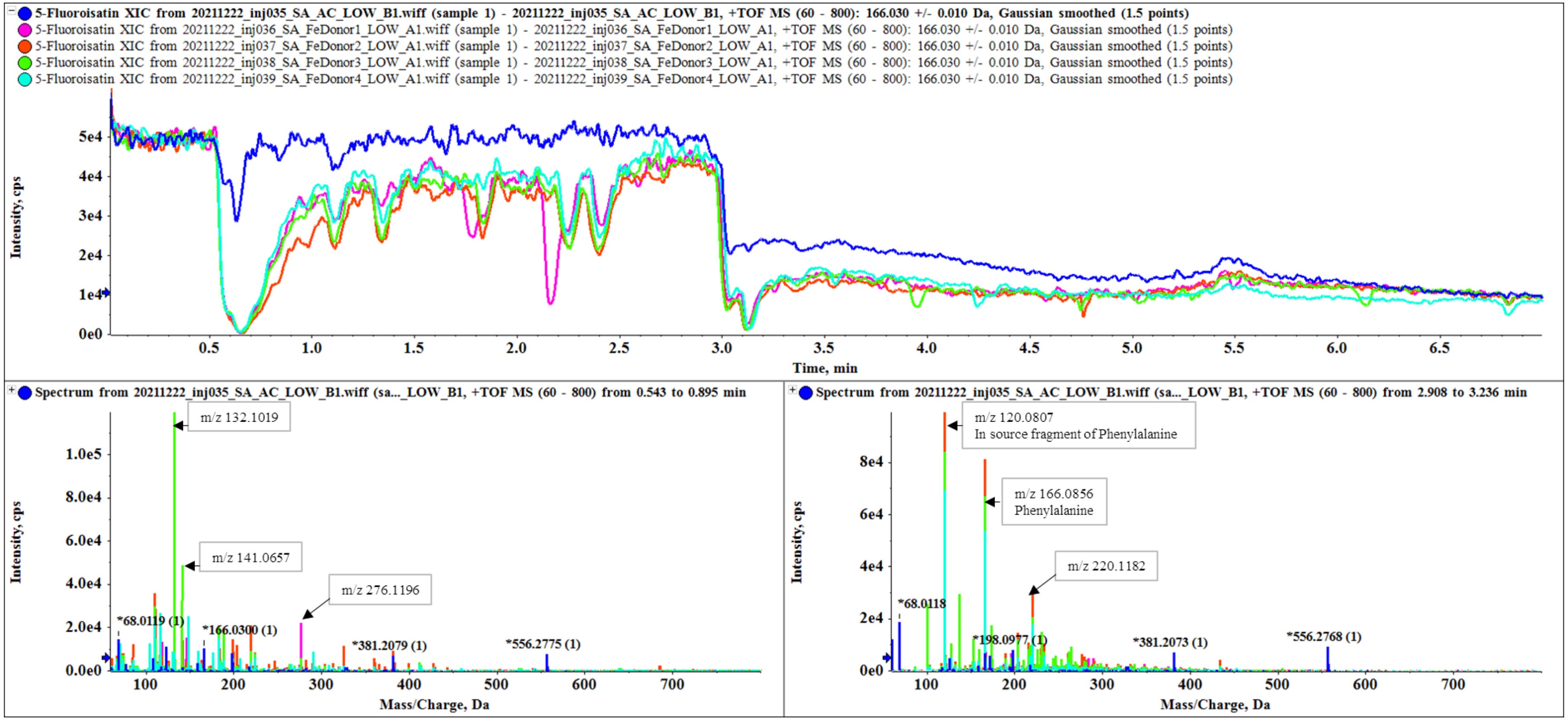

Figure S16. Mass spectrum inspection of the suppressed areas in fecal samples with 5-fluoroisatin in positive mode

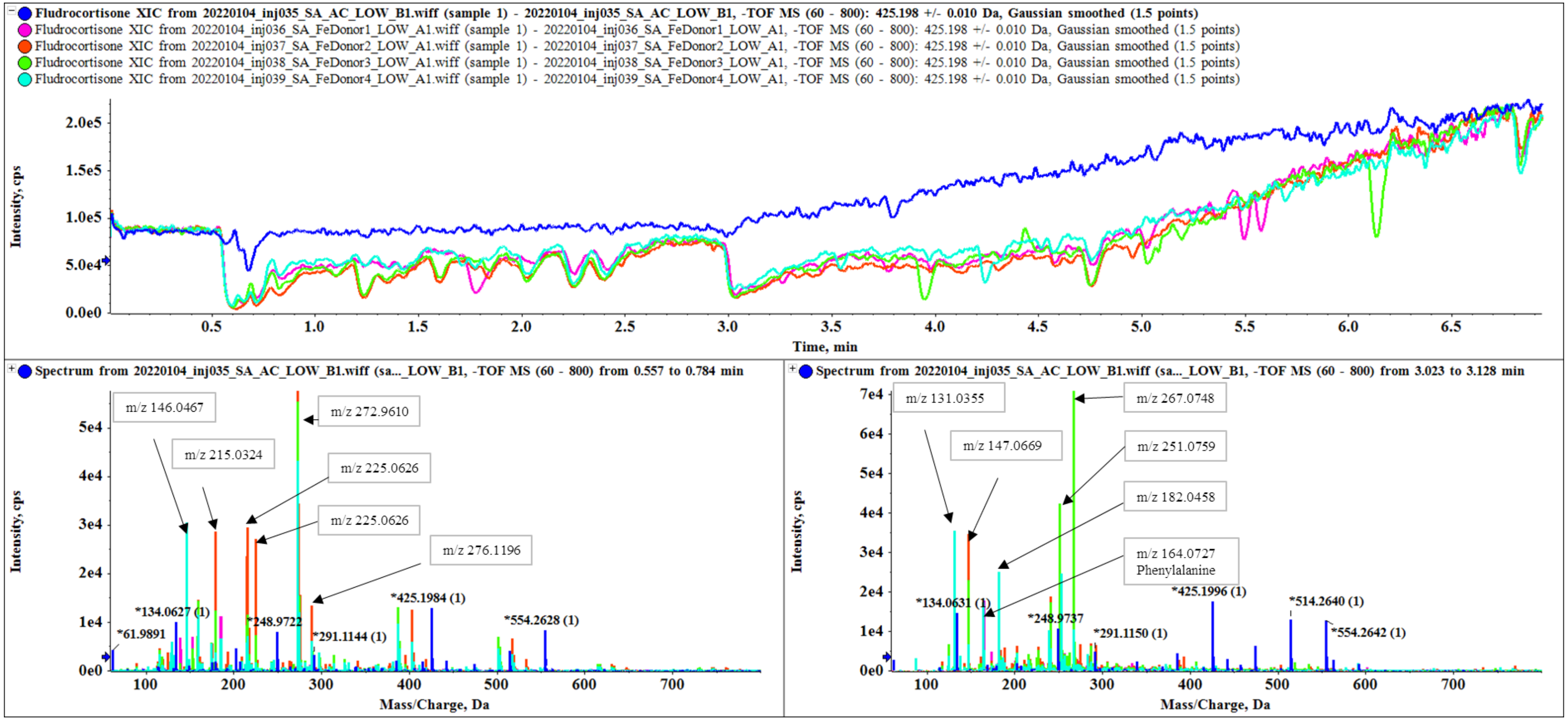

Figure S17.Mass spectrum inspection of the suppressed areas in fecal samples with fludrocortisone in negative mode
